# Chemically Tailored Growth of 2D Semiconductors via Hybrid Metal–Organic Chemical Vapor Deposition

**DOI:** 10.1021/acsnano.4c02164

**Published:** 2024-09-04

**Authors:** Zhepeng Zhang, Lauren Hoang, Marisa Hocking, Zhenghan Peng, Jenny Hu, Gregory Zaborski, Pooja D. Reddy, Johnny Dollard, David Goldhaber-Gordon, Tony F. Heinz, Eric Pop, Andrew J. Mannix

**Affiliations:** † Department of Materials Science & Engineering, Stanford University, Stanford, California 94305, United States; ‡ Department of Electrical Engineering, Stanford University, Stanford, California 94305, United States; § Department of Applied Physics, Stanford University, Stanford, California 94305, United States; ∥ Department of Physics, Stanford University, Stanford, California 94305, United States; ⊥ Stanford Institute for Materials and Energy Sciences, SLAC National Accelerator Laboratory, Menlo Park, California 94025, United States; # Department of Photon Sciences, Stanford University, Stanford, California 94305, United States; ¶ Precourt Institute for Energy, Stanford University, Stanford, California 94305, United States

**Keywords:** metal−organic chemical vapor deposition, 2D semiconductor growth, transition-metal dichalcogenides, doping, alloy, WS_2_, MoS_2_

## Abstract

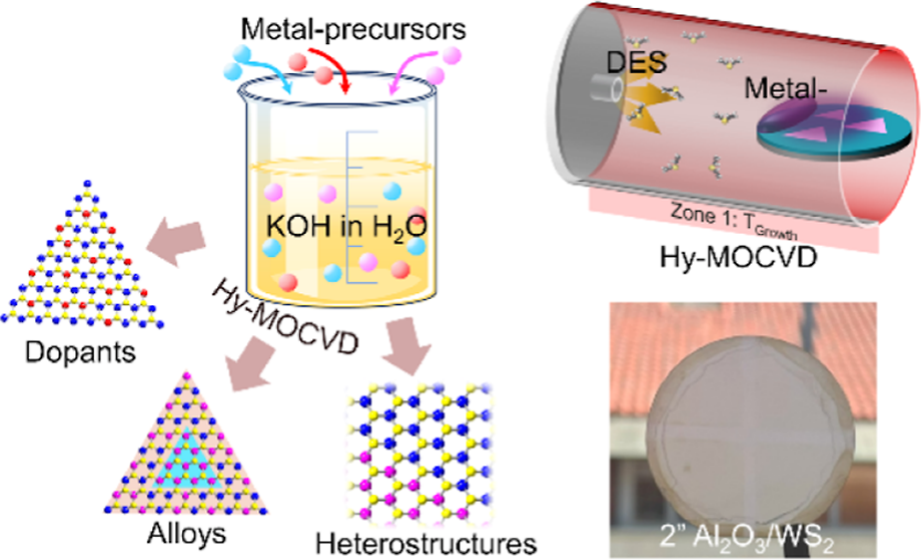

Two-dimensional (2D) semiconducting transition-metal dichalcogenides (TMDCs) are an exciting platform for excitonic physics and next-generation electronics, creating a strong demand to understand their growth, doping, and heterostructures. Despite significant progress in solid-source (SS-) and metal–organic chemical vapor deposition (MOCVD), further optimization is necessary to grow highly crystalline 2D TMDCs with controlled doping. Here, we report a hybrid MOCVD growth method that combines liquid-phase metal precursor deposition and vapor-phase organo-chalcogen delivery to leverage the advantages of both MOCVD and SS-CVD. Using our hybrid approach, we demonstrate WS_2_ growth with tunable morphologies—from separated single-crystal domains to continuous monolayer films—on a variety of substrates, including sapphire, SiO_2_, and Au. These WS_2_ films exhibit narrow neutral exciton photoluminescence line widths down to 27–28 meV and room-temperature mobility up to 34–36 cm^2^ V^–1^ s^–1^. Through simple modifications to the liquid precursor composition, we demonstrate the growth of V-doped WS_2_, Mo_*x*_W_1–*x*_S_2_ alloys, and in-plane WS_2_–MoS_2_ heterostructures. This work presents an efficient approach for addressing a variety of TMDC synthesis needs on a laboratory scale.

Two-dimensional (2D) semiconducting transition-metal dichalcogenides (TMDCs), such as monolayer MoS_2_, WS_2_, and WSe_2_, have emerged as attractive candidates for next-generation electronics due to their atomic-scale thickness, tunable band structure, and excellent electronic properties.^1−3^ In the past decade, demonstrations of high-performance 2D TMDC-based transistors, optoelectronics, and logical circuits have escalated demand for the accurately controlled large-area growth of high-quality pure and p-/n-type-doped 2D TMDC monolayers.^4−12^ Solid source chemical vapor deposition (SS-CVD) has become a popular approach for growing 2D TMDCs in laboratory settings due to its low equipment cost, flexibility, and rapid growth, enabling efficient optimization. By using SS-CVD, a wide range of 2D TMDCs, such as MoS_2_,^13,14^ WS_2_,^6^ V-doped WSe_2_,^9,10^ Fe-doped MoS_2_,^15^ and Mo_*x*_W_1–*x*_S_2_ alloys^16^ have been successfully synthesized, and wafer-scale TMDC synthesis and device fabrication have been demonstrated.^5,17^

However, further optimization for SS-CVD growth is necessary and challenging. For example, solid sources typically exhibit low sublimation rates and poor sublimation stability during the material growth process. The solid precursor is challenging to replenish midgrowth, resulting in variable stoichiometry in the reactor over time during each growth run. Small variations in the source amount and position modify the uniformity of the growth. These factors limit the tolerance and controllability of SS-CVD.^18,19^ Moreover, although a specific SS-CVD strategy normally works well for an individual TMDC system, a universal method for multiple-material synthesis remains underdeveloped. Even though the situation has been improved by source supply strategies^20,21^ and adding promoters,^22−24^ the design of state-of-the-art SS-CVD growth setups has also become increasingly complex—and, correspondingly, less accessible—for most laboratory research.

On the other hand, metal–organic CVD (MOCVD) has shown good reproducibility and large-area uniformity in 2D TMDC growth^25^ at relatively low reaction temperatures (150–320 °C)^26−28^ and under accurate precursor control due to the use of vapor phase metal–organic metal (M-organic) and hydride or organic chalcogen (X-organic) precursors.^25^ However, to reduce carbon impurity incorporation, MOCVD often uses low precursor concentrations, resulting in slow growth rates of the 2D TMDCs. Moreover, each dopant metal–organic source requires a separate precursor supply line in the MOCVD system to avoid cross-contamination, which increases the system cost and complexity and hinders the exploration of substitutional doping. Alkali metal-based solid and gas phase growth promoters have been explored in MOCVD to increase the growth rate and decrease the nucleation density.^29−31^ However, several potentially negative effects have been reported from alkali metal salts used in MOCVD, including disruption of epitaxy, the introduction of nanoscale particles, and degradation of optical and electronic properties.^32^ Consequently, further research and optimization are crucial to understanding the mechanisms and optimize the use of growth promoters in MOCVD. Despite the development of MOCVD strategies to enlarge the domain size,^33^ enable epitaxy,^34^ and reduce the growth temperature,^26,27^ more accessible and efficient MOCVD growth and doping methods are still needed.

Here, we report a hybrid MOCVD (Hy-MOCVD) growth method that delivers metal precursors and growth promoters from the solution phase and metal–organic chalcogen precursors from the vapor phase, to combine the advantages of both MOCVD and SS-CVD and realize efficient growth of multiple types of 2D TMDCs. Aqueous Hy-MOCVD precursor delivery by both spin-coating and dip-coating produces WS_2_ monolayers with good controllability and uniformity. Hy-MOCVD grown WS_2_ exhibits typical domain sizes of tens of micrometers, good optical quality with room temperature neutral exciton peak width down to 27–28 meV, good electronic performance with electron mobility up to 34–36 cm^2^ V^–1^ s^–1^, and transistor on/off ratio of >10^7^. Hy-MOCVD also enables the growth of WS_2_ on diverse substrates, such as *c*-plane and *a*-plane sapphire, Si/SiO_2_, and sapphire/Au. To illustrate the versatility of our Hy-MOCVD approach, we also demonstrate the facile growth of V-doped WS_2_, Mo_*x*_W_1–*x*_S_2_ alloys, and WS_2_–MoS_2_ heterostructures without any modifications to the growth hardware. Compared with alkali metal-assisted MOCVD,^29−32^ Hy-MOCVD not only yields similar benefits of increased grain size and suppressed multilayer nucleation but also provides an effective strategy for engineering the growth promoter concentration, transition metal dopants, alloy composition, and heterostructures of TMDCs on versatile substrates for a wide range of academic research.

## Results and Discussion

**Figure 1:**
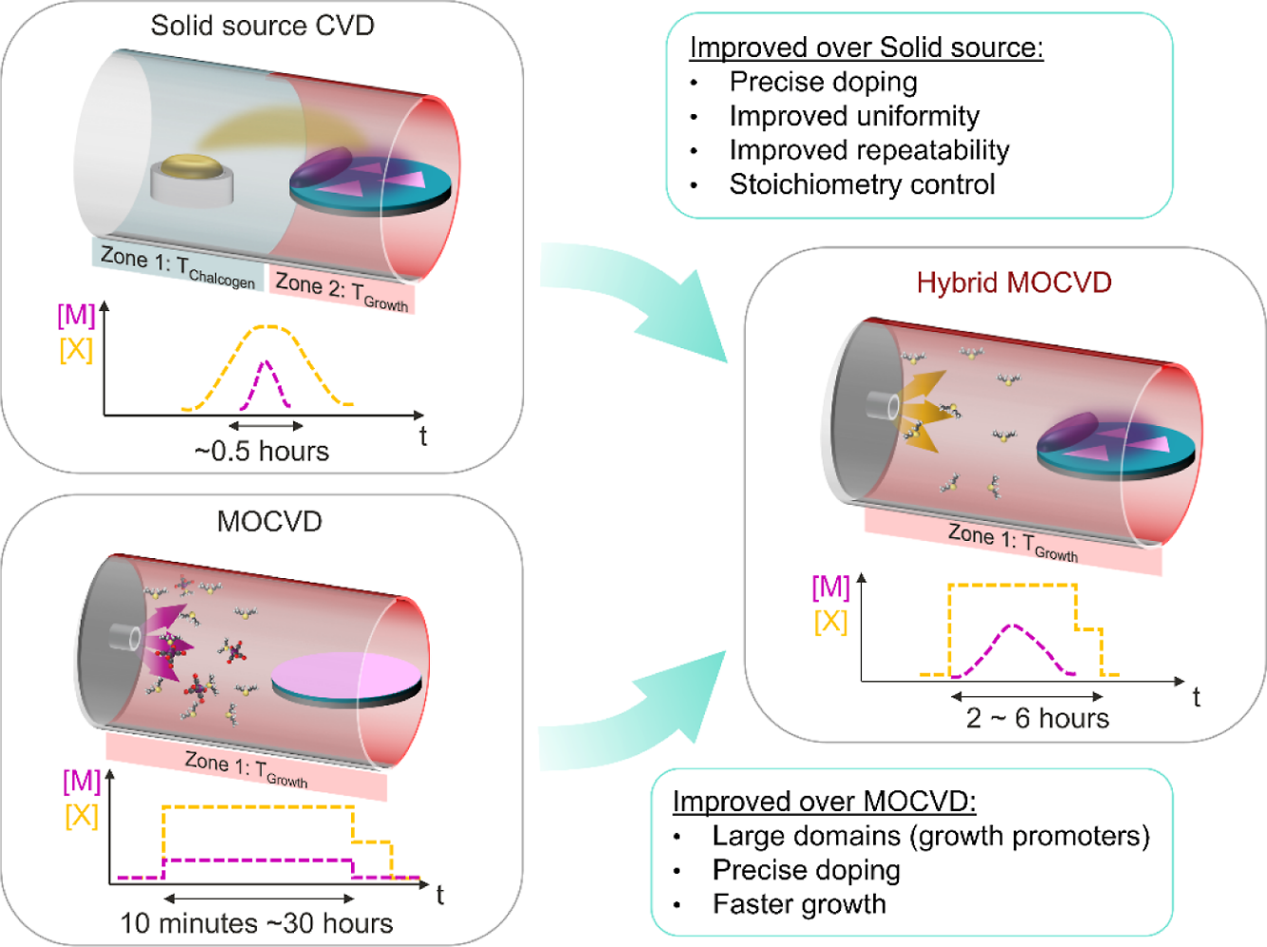
Principles of Hy-MOCVD. Representative schematics of the growth setups and precursor supply time profiles for conventional SS-CVD, MOCVD, and Hy-MOCVD. The *y*-axis in the time profiles stands for the active concentrations of the transition metal (M) and chalcogen (X) species. The MOCVD growth time can vary widely due to differences in growth temperature, heating methods, growth promoters, and precursor flow rates employed by various groups (as summarized in Table S2).

In Figure 1, we compare the concepts and strengths of SS-CVD, MOCVD, and Hy-MOCVD. The Hy-MOCVD method employs both X-organic precursors used in MOCVD and inorganic transition metal precursors (M-inorganic) used in SS-CVD. As in MOCVD, the X-organic precursor was introduced into the Hy-MOCVD chamber in the vapor phase via a bubbler and a mass flow controller (see Figure S1 for the setup schematic of Hy-MOCVD). This ensures a stable chalcogen concentration throughout the entire growth process, which is necessary for stoichiometrically controlled growth. Precise combinations of the primary transition metal element(s), substitutional dopants, and any growth promoter species are more challenging to deliver due to their lower vapor pressure, yet these are also critical to the outcome of the growth process.^23,26^ To overcome the uncontrolled flux of SS powders and the expense of metal–organic precursor delivery, M-inorganic precursors with growth promoter KOH were deposited onto the growth substrate by aqueous solution coating before Hy-MOCVD growth. This localized transition metal supply ensures a high concentration of reactive M species on the wafer surface during growth. Moreover, by mixing M-inorganic and KOH with other dopant sources,^8,9,35,36^ Hy-MOCVD can be used for the growth of doped TMDCs and TMDC alloys with extreme precision via dilution.^12,36^ Summarizing these advantages (Table S1), Hy-MOCVD combines the precise control over chalcogen stoichiometry found in MOCVD with the versatility and efficiency in switching or mixing transition metals and growth promoters offered by SS-CVD. In the following sections, we will demonstrate these advantages by using Hy-MOCVD to grow WS_2_ and incorporate dopants, alloys, and heterostructures.

**Figure 2:**
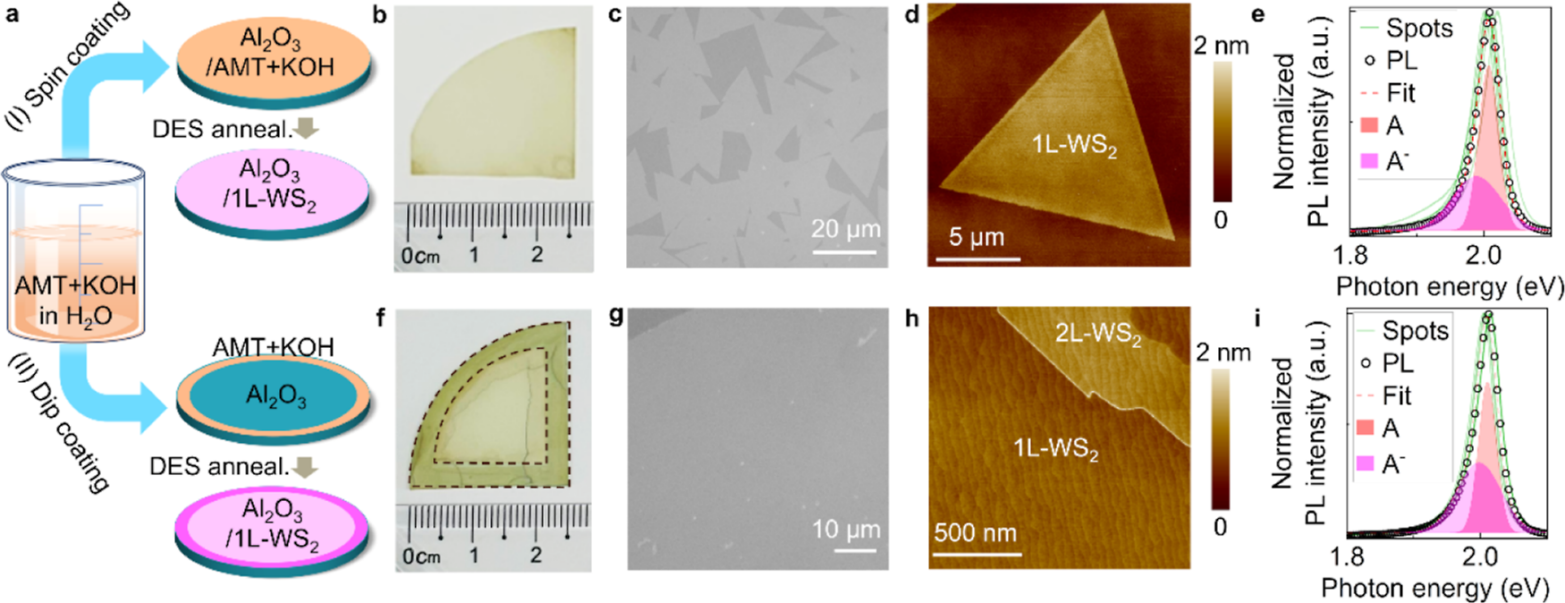
Hy-MOCVD processes. (a) Schematics of the two paths to Hy-MOCVD: (I) spin-coating and (II) dip-coating. (b–e) Characterization of representative WS_2_ film synthesized via spin-coating (Path I), consisting of (b) photograph of *c*-plane sapphire/WS_2_ wafer, (c) contrast-enhanced optical microscope image, (d) AFM topography image, and (e) normalized photoluminescence (PL) spectra collected from 8 random spots on spin-coating Hy-MOCVD monolayer WS_2_, including overlaid Gaussian peaks fit to the narrowest spectrum. (f–i) Characterization of representative WS_2_ synthesized via dip-coating (Path II), consisting of: (f) photograph, (g) optical micrograph, (h) AFM topography image, and (i) normalized PL spectra collected from 8 random spots, with overlaid fit to narrowest spectrum. Overlaid dashed lines in (f) highlight the dip-coated area on the edges of *c*-plane sapphire wafer.

In the Hy-MOCVD growth of WS_2_, diethyl sulfide (DES, (CH_3_CH_2_)_2_S) and ammonium metatungstate hydrate (AMT, (NH_4_)_6_H_2_W_12_O_40_·*x*H_2_O) were used as the X-organic and M-inorganic precursors, respectively. Delivery of the metal solution to the substrate is flexible, and we explored two paths in this work: spin- and dip-coating (Figure 2a). In spin-coating delivery, the starting solution of AMT and KOH in deionized (DI) water was spin coated onto a UV–ozone-treated wafer, and the water was removed by heating at 80 °C in air. The coated wafer was then transferred to the tube furnace MOCVD system and annealed in a DES vapor environment (0.05–0.12 sccm) at 775 °C for 2–6 h to conduct the growth. Photographs of a typical WS_2_ on *c*-plane sapphire wafer after the growth show a uniform color across the wafer (Figure 2b). Optical microscopy images show homogeneous coverage of WS_2_ triangular domains, typically ∼20 μm in width, with sharp and straight edges (Figure 2c). Atomic force microscopy (AFM) shows the monolayer thickness and clean surface of Hy-MOCVD grown WS_2_ (Figures 2d and S2). Typical photoluminescence (PL) spectra show strong and narrow neutral exciton peaks (A) at 2.01 eV with the narrowest full width at half-maximum (fwhm) of 28 meV (Figures 2e and S3), indicating the good quality of Hy-MOCVD grown WS_2_.^37,38^ The lower-energy shoulder peak is attributed to the negatively charged exciton (A^–^), consistent with the n-type electronic transport characteristics observed in Hy-MOCVD WS_2_ monolayers, as discussed in a later section.

In dip-coating delivery, the *c*-plane sapphire wafer edges were dipped into an aqueous solution of AMT and KOH. As with the spin-coating path, the dip-coated wafer was then dried in air at 80 °C, and annealed in DES. During the growth process, reactive species diffuse from the highly concentrated AMT + KOH sources at the sample edges, triggering the growth of WS_2_ on the uncoated center area of the wafer. Typical photos of the wafer show deeper color on the dip-coated edges and uniform light yellow-green in the center of the wafer (Figure 2f). An optical micrograph taken from the center of the wafer shows a continuous WS_2_ film with small multilayer islands (Figure 2g). AFM images acquired around a multilayer island show well-defined single-layer-height steps of the bilayer island and clear atomic steps and terraces of the *c*-plane sapphire substrate visible through the monolayer, indicating the clean surface of the WS_2_ film (Figure 2h). PL spectra collected from continuous monolayer regions of these samples typically show A exciton peaks centered at 2.01 eV (with the narrowest fwhm of 27 meV), consistent with a good-quality monolayer film (Figures 2i and S3).^37^ We have found that both spin-coating and dip-coating yield good-quality and consistent growth. Using X-ray photoelectron spectroscopy (XPS), we detected trace signatures for residual K following Hy-MOCVD growth on WS_2_ samples grown using both spin-coating and dip-coating precursor delivery. We observe that this signal is removed during wet transfer processes (Figure S4a), which suggests that the residual K species are not incorporated within the WS_2_ lattice. Dip-coating Hy-MOCVD can grow continuous monolayer WS_2_ on sapphire on demand over a long period up to 17 months, showcasing the excellent repeatability of Hy-MOCVD (Figure S5). To demonstrate that Hy-MOCVD can be broadly applied to other TMDCs, we grew monolayer MoS_2_ and WSe_2_ with dip-coating Hy-MOCVD (Figure S6).

**Figure 3:**
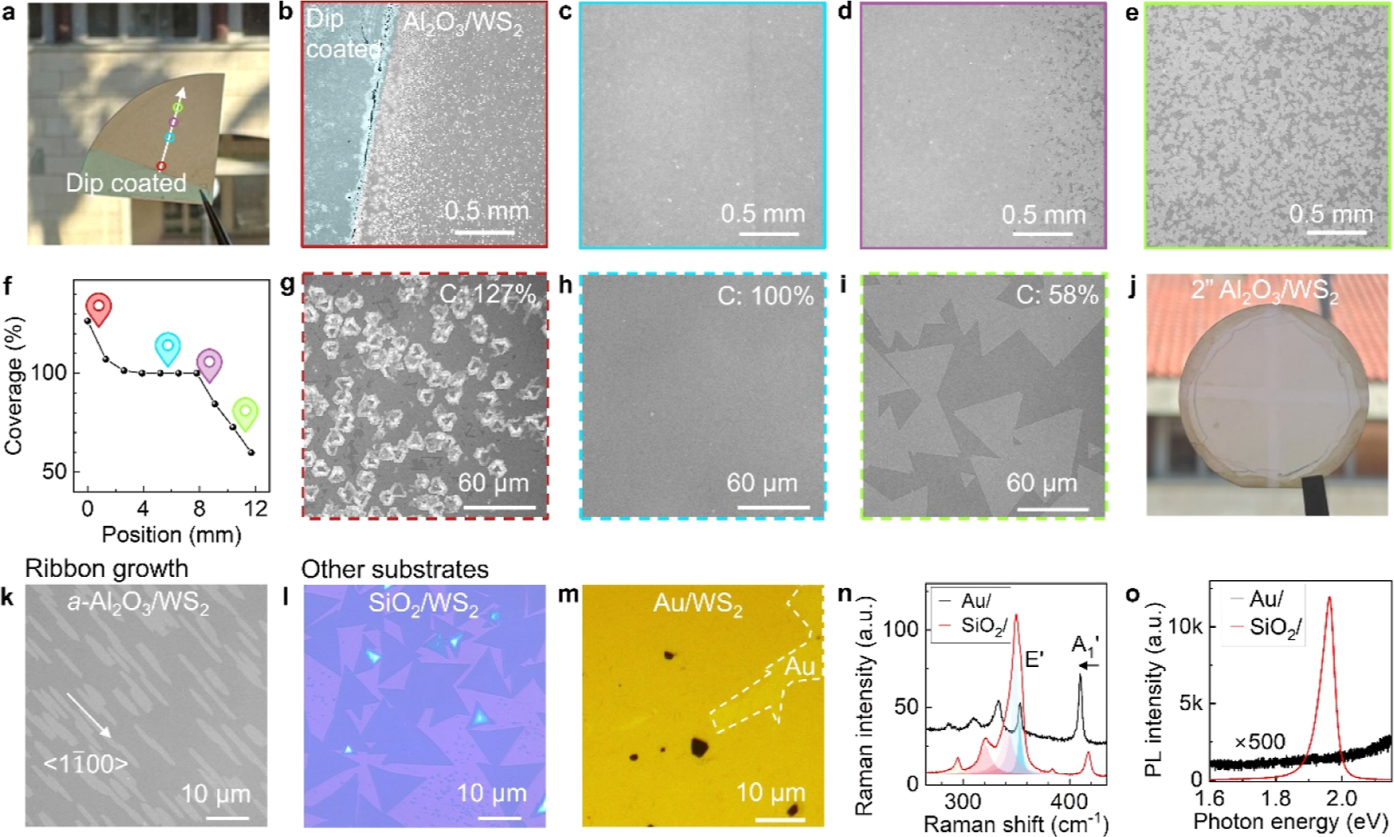
Morphology control and compatibility with other substrates. (a) Photo of Hy-MOCVD grown *c*-plane sapphire/WS_2_ wafer, with a single edge dip-coated by precursor solution. (b–e) Contrast-enhanced optical images of sapphire/WS_2_ taken from the locations highlighted by colored circles in (a). (f) WS_2_ coverage versus position along the arrow in (a). Positions of (b–e) are highlighted with corresponding colors. (g–i) Contrast-enhanced zoom-in optical images of multilayer, continuous monolayer, and noncontinuous monolayer regions. *C* stands for the coverage extracted from the corresponding image. (j) Photo of Hy-MOCVD grown WS_2_ on a 2″ *c*-plane sapphire wafer via dip-coating. (k) Optical image of Hy-MOCVD grown WS_2_ ribbons on annealed *a*-plane sapphire with 1° miscut angle toward *c*-plane. (l) Optical image of Hy-MOCVD WS_2_ grown on Si/SiO_2_ substrate. (m) Optical image of Hy-MOCVD grown WS_2_ grown on sapphire/Au substrate. (n,o) Raman and PL spectra of WS_2_ grown on SiO_2_ and Au substrates, respectively.

Growth producing a well-defined compositional gradient can be valuable for exploratory synthesis. Dip-coating Hy-MOCVD can exploit the vapor-phase transport gradient to grow WS_2_ with different morphologies and high compatibility with different substrates. Figure 3a shows a photograph of the *c*-plane sapphire wafer after Hy-MOCVD growth with only one edge coated with AMT + KOH solution. The WS_2_ coverage changes with increasing distance from the dip-coating boundary (Figure 3b–e), with a typical profile given by Figure 3f (extracted from binary thresholding of microscope images; coverage over 100% indicates multilayer islands over a continuous monolayer film). At higher magnification within these regions, we observed that WS_2_ grew as a continuous film with a high density of multilayer islands in the area close to the dip-coating boundary (Figure 3g). This converts to a continuous monolayer with a low density of multilayer islands in the center of the wafer (Figure 3h) and finally becomes isolated domains on the far end (Figure 3i). The high coverage region (>70%) of predominantly monolayer WS_2_ extends to approximately 1 cm away from the dip-coating metal source region, which is typical of samples grown in this way. Ozone etching reveals the grain boundaries^39^ within the continuous WS_2_ regions (Figure S7), and we observe that the average WS_2_ domain size varies from 3 to 30 μm with increasing distance from the dip-coating boundary.

As shown in Figure 3j, dip-coating can be applied to enable Hy-MOCVD growth across a 2″ *c*-plane sapphire wafer. The coverage and uniformity near the wafer center were improved by dip-coating the wafer edge and placing two crossed AMT + KOH dip-coated W foil strips on the substrate. This setup increases the local flux of W-species near the wafer center. The uniformity of Hy-MOCVD growth across the 2″ sapphire wafer was evaluated by Raman mapping (Figure S8), which demonstrated that the WS_2_ film grown on the bare sapphire area is primarily monolayer, with an average 2LA + E′ to A_1_′ peak distance of 65.4 ± 0.8 cm^–1^, and exhibits a crystalline quality similar to SS-CVD, with an average A_1_′ peak width of 5.0 ± 0.5 cm^–1^.^6,40^ The narrow distributions of both metrics confirm the uniformity of Hy-MOCVD WS_2_.

Growth on multiple substrates is important for the laboratory-scale optimization and integration of TMDCs. Hy-MOCVD growth of WS_2_ on annealed *a*-plane sapphire substrates with 1° miscut angle toward the *c*-plane (Figure 3k) resulted in WS_2_ ribbons oriented along the substrate ⟨11̅00⟩ terrace edge direction (see Figure S9 for the AFM images). This morphology is consistent with previous observations of epitaxial growth of MoS_2_ and WS_2_ on *a*-plane and vicinal *a*-plane sapphire via SS-CVD,^7,41^ which is attributed to the anisotropic growth induced by the 2-fold symmetry *a*-plane sapphire lattice. Polarization-resolved second-harmonic generation (SHG) reveals that the Hy-MOCVD grown WS_2_ ribbons exhibit predominantly two sets of epitaxial lattice orientations, with the WS_2_ armchair directions oriented parallel to either the ⟨1–100⟩ or ⟨0001⟩ directions of the *a*-plane sapphire (Figure S10). However, this epitaxial behavior is different from the unidirectional epitaxial growth of MoS_2_ and WS_2_ on *a*-plane sapphire and vicinal *a*-plane sapphire, which can be attributed to the difference between the substrate miscut angle, substrate annealing conditions, and growth chemistry. Previous studies have reported that the use of alkali metal salts can have an impact on epitaxial behavior as well.^32^ Our results suggest that Hy-MOCVD can realize van der Waals epitaxial growth of 2D TMDCs and can be used for understanding how precursors and alkali metal-based growth promoters modify epitaxy. Additionally, Hy-MOCVD is compatible with the growth of WS_2_ on standard thermally oxidized Si/SiO_2_ substrates and on Au thin films deposited on *c*-plane sapphire substrates (Figure 3l,m). Notably, the Raman out-of-plane mode (A_1_′) of WS_2_ on Au exhibits a redshift of ∼7 cm^–1^, shifting the peak center to 410 cm^–1^, while the in-plane mode (E′) remains unaltered at ∼354 cm^–1^ compared to WS_2_ grown on SiO_2_ (Figure 3n). This observation aligns with the reported A_1_′ mode downshifting in exfoliated WS_2_ monolayer on Au and suggests a strong interaction between monolayer WS_2_ and Au.^42^ PL of WS_2_ grown on Si/SiO_2_ confirms its high quality, whereas the quenched PL for WS_2_ grown on Au indicates nonradiative transition dominated recombination of excitons in the Au/WS_2_ stack (Figure 3o). Furthermore, Hy-MOCVD WS_2_ on different substrates exhibited an absence of Raman peaks within the 1300–1600 cm^–1^ range (see Figure S11 for the Raman spectra), indicating that the films are free of amorphous carbon. Figure S4b presents a comparison between C 1s core-level spectra for Hy-MOCVD WS_2_ and those of the bare substrate, which confirms the absence of carbon deposition during the growth process.

**Figure 4:**
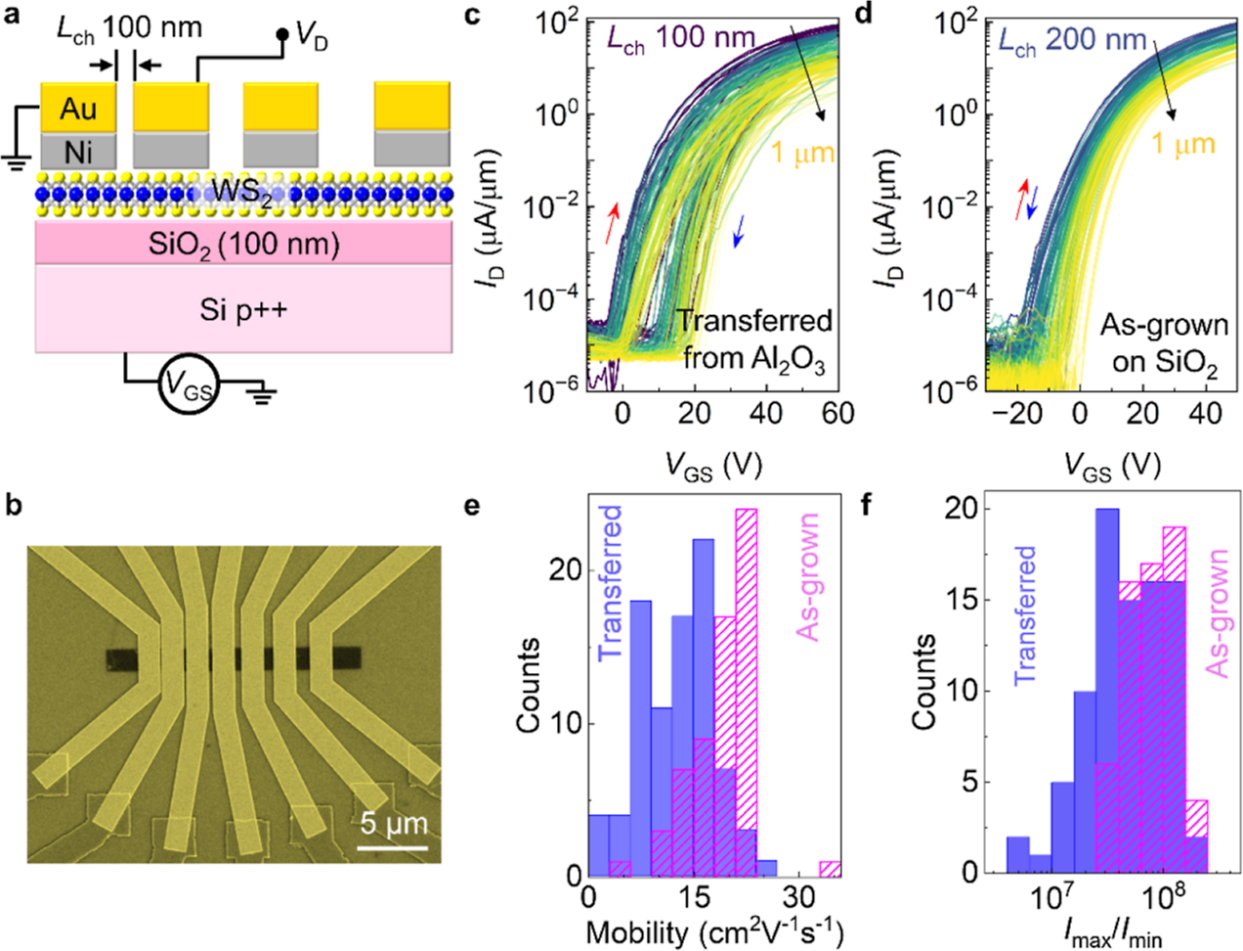
Electrical characteristics of monolayer WS_2_ grown by Hy-MOCVD. (a) Schematic of a back-gated transistor based on Hy-MOCVD WS_2_. (b) False color SEM image of WS_2_-based TLM device. (c) Measured *I*_D_ vs *V*_GS_ curves for FETs of transferred Hy-MOCVD WS_2_ with designed channel length *L*_ch_ of 100, 200, 300, 500, 700, and 1000 nm, from purple to yellow at *V*_DS_ = 1 V. Red and blue arrows represent the forward and backward *V*_GS_ sweeping directions, respectively. (d) Measured *I*_D_ vs *V*_GS_ curves for FETs of as-grown Hy-MOCVD WS_2_ with designed channel length *L*_ch_ of 200, 300, 500, 700, and 1000 nm (from blue to yellow). Red and blue arrows represent the forward and backward *V*_GS_ sweeping directions, respectively. Histograms of measured (e) field-effect mobility and (f) *I*_max_/*I*_min_ for FETs of transferred and as-grown Hy-MOCVD WS_2_ (extracted from forward *V*_GS_ sweeps).

To evaluate the electronic properties of Hy-MOCVD grown WS_2_, we fabricated back-gated field-effect transistors (FETs) through two processes: by transferring the Hy-MOCVD monolayer WS_2_ from sapphire onto SiO_2_ (100 nm) on highly doped p^++^ Si and by using as-grown Hy-MOCVD monolayer WS_2_ directly on similar substrates. FET channel regions (100 nm to 1 μm) were defined by electron-beam lithography on WS_2_ triangular domains and contacted with Ni/Au electrodes to achieve transfer length method (TLM) structures (Figure 4a,b).^43^ Measured drain current vs back-gate voltage (*I*_D_ vs *V*_GS_) characteristics of such WS_2_ FETs exhibit consistent n-type behavior across 10–17 devices for each channel length, illustrating the uniformity of Hy-MOCVD grown WS_2_ (Figure 4c,d).

The devices with transferred WS_2_ exhibit maximum electron mobility between 24 and 33 cm^2^ V^–1^ s^–1^ (this value is given as a range of two numbers, extracted from the forward and backward sweeps, due to the observed clockwise hysteresis), with an average value between 13 and 18 cm^2^ V^–1^ s^–1^ and median value between 13 and 19 cm^2^ V^–1^ s^–1^. We see a notable average *I*_max_/*I*_min_ ratio of 10^7^ (Figure 4e,f). The shortest devices with a 100 nm channel length have a good on-state current density, reaching a maximum value of 88 μA/μm and an average of 65 μA/μm at *V*_DS_ = 1 V (see Figure S12a for the *I*_D_ versus channel length plot). These metrics surpass those of most SS-CVD and MOCVD-grown monolayer WS_2_-based FETs with similar configurations, indicating the good quality of Hy-MOCVD WS_2_ (Table S3 for a device performance comparison). The contact resistance can lead to errors in the field-effect mobility estimate, especially in shorter channel length devices (Figure S12c shows field-effect mobility versus channel length). The device performance can potentially be improved by incorporating lower resistance contacts and high-κ dielectric layers.^44−47^ FET devices fabricated from Hy-MOCVD WS_2_ grown directly on the Si/SiO_2_ substrate exhibit improved field-effect mobility with a maximum between 34 and 36 cm^2^ V^–1^ s^–1^, an average value of 19–21 cm^2^ V^–1^ s^–1^, a median value of 20–22 cm^2^ V^–1^ s^–1^ (Figure 4e), and less *I*_D_ hysteresis for forward-to-backward *V*_DS_ sweeps (Figures 4c,d and S12c–h). This suggests that the performance of Hy-MOCVD grown on sapphire substrates is limited by either transfer-induced damage (see broadened Raman and PL peaks of WS_2_ after the transfer in Figure S13) or a difference in crystal quality versus growth on Si/SiO_2_ substrates.

**Figure 5:**
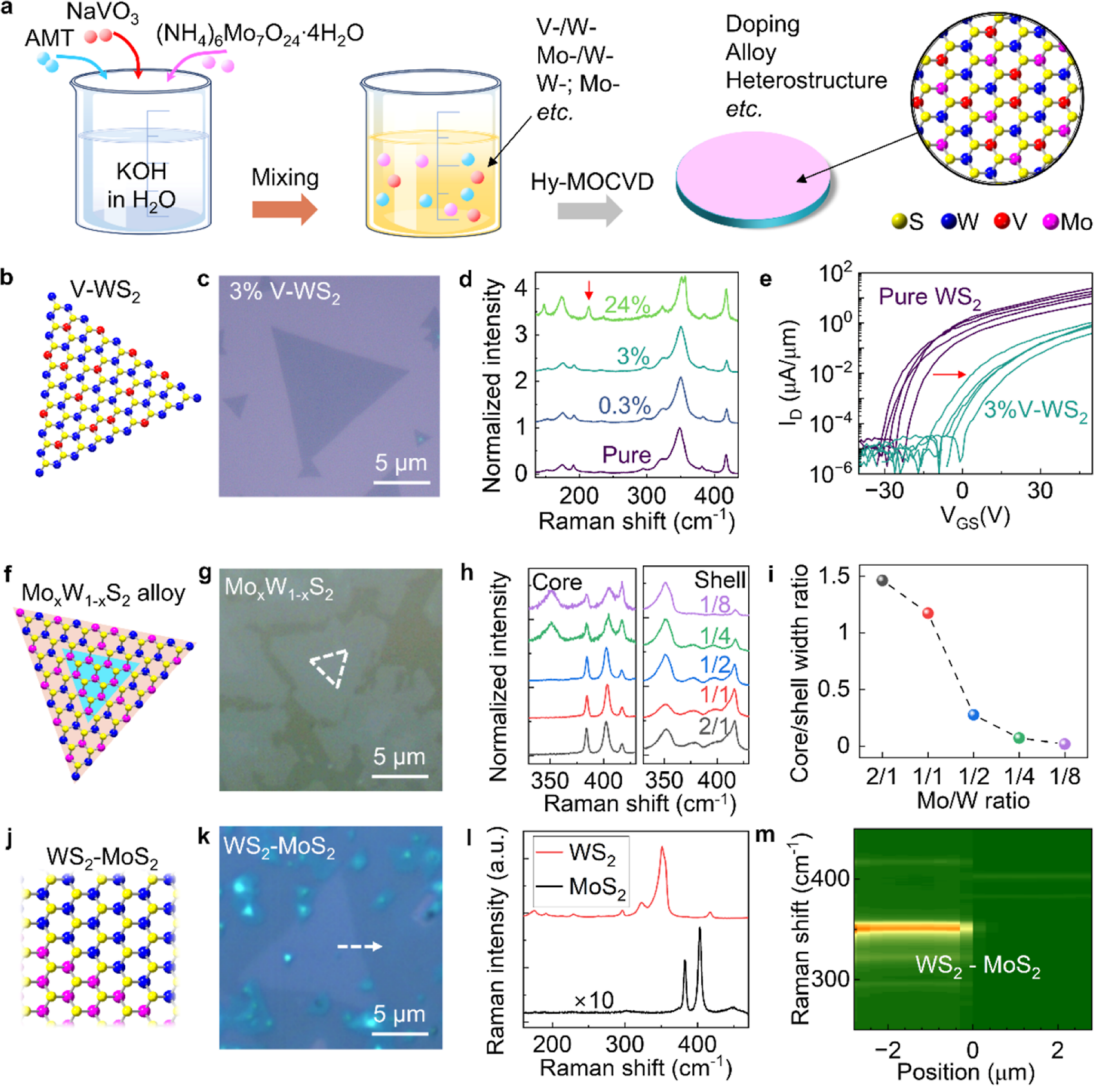
Transition metal engineering of monolayer WS_2_ using Hy-MOCVD. (a) Schematic of transition metal engineering of monolayer WS_2_ using Hy-MOCVD. (b) Lattice schematic of V-doped WS_2_. (c) Optical image of as-grown Hy-MOCVD V-doped WS_2_ on Si/SiO_2_ substrate with a nominal doping concentration of 3%. (d) Typical Raman spectra of V-doped WS_2_ with different nominal doping ratios of 0, 0.3, 3, and 24%. (e) Measured *I*_D_ vs *V*_GS_ curves for monolayer undoped WS_2_ and V-doped WS_2_ FET devices with channel length of 500 nm and *V*_DS_ = 1 V. (f) Lattice schematic of in-plane MoS_2_–Mo_*x*_W_1–*x*_S_2_ heterostructure with a MoS_2_ core and a Mo_*x*_W_1–*x*_S_2_ alloy shell. (g) Optical image of Hy-MOCVD grown in-plane MoS_2_–Mo_*x*_W_1–*x*_S_2_ heterostructure on *c*-plane sapphire substrate. MoS_2_ core is circled with a white dashed line. (h) Typical Raman spectra of Mo_*x*_W_1–*x*_S_2_ alloy core (left) and shell (right) grown with different Mo/W mole ratios in the starting solution of Hy-MOCVD. (i) Core/shell width ratio versus Mo/W mole ratio of starting solution. (j) Lattice schematic of Hy-MOCVD grown WS_2_–MoS_2_ in-plane heterostructure. (k) Optical image of Hy-MOCVD grown WS_2_–MoS_2_ in-plane heterostructure. (l) Typical Raman spectra collected from the two sides of WS_2_–MoS_2_ in-plane heterostructure. (m) Raman spectra line scan along the arrow in (k).

Directly incorporating dopants into TMDCs and growing TMDC alloys and heterostructures from synthesis have sparked substantial interest. Hy-MOCVD enables convenient adjustment of the TMDC metal composition based on the precise addition of various water–soluble transition metal sources to the precursor solution (Figure 5a). V-doped WS_2_ monolayers with a nominal doping from 0.3 to 24% (V/(V + W) atom mole ratio in the precursor solution) were grown on Si/SiO_2_ substrates (Figure 5b,c) by adding sodium metavanadate (NaVO_3_) into the AMT + KOH precursor solution. The emergence of a Raman mode at around 213 cm^–1^ in nominal 24% V-doped WS_2_, and the decrease of the 2LA(M) + E′ peak intensity with the increase of the nominal doping ratio, are consistent with previous V-WS_2_ literature (see Figures 5d and S14 for nominal doping ratio dependence of 2LA + E′ peak intensity).^9,48^ The characteristic peak at 213 cm^–1^ can be assigned to the multiphonon mode of E″(M)–TA(M), suggesting that V is substitutionally incorporated into WS_2_.^48^ Transistors fabricated using the 3% V-doped WS_2_ exhibit a threshold voltage shift of +23 V compared with undoped WS_2_ devices (Figure 5e), on the 100 nm SiO_2_ back-gate insulators. This is consistent with the expected p-type doping from substitutional V acceptors in the TMDC monolayer.^9,36^ Additional optimizations of doping concentration and FET metal contacts are needed to achieve a hole current. XPS characterization (Figure S15) shows the measured V/(V + W) atom ratio increasing monotonically with nominal doping concentration, accompanied by shifting of the W 4f and S 2p core levels toward lower binding energy as expected for a p-type dopant. We also demonstrated Re doping in Hy-MOCVD. Compared with pure WS_2_, we found that the PL emission is evidently quenched in both V-doped and Re-doped WS_2_ (Figure S16). These observations are consistent with previous reports of quenched PL in doped samples, including V-WS_2_, Re-WSe_2_, V-MoS_2_, and Re-MoS_2,_^9,11,49^ where the PL quenching can be attributed to the in-gap dopant state-mediated exciton recombination and/or additional charge carriers.^12,49^

Hy-MOCVD similarly enables alloy and heterostructure growth. We grew Mo_*x*_W_1–*x*_S_2_ alloys exhibiting an in-plane heterostructure with a core and a shell of different alloy compositions during a single-step dip-coating Hy-MOCVD growth (Figure 5f,g) by mixing ammonium molybdate ((NH_4_)_6_Mo_7_O_2_·4H_2_O) into the AMT + KOH solution. We provide additional confirmation of the core–shell compositional variation via fluorescence imaging of the MoS_2_ and WS_2_ PL emission on a transferred alloy sample in Figure S17a,b and XPS characterization, which shows the coexistence and splitting of the Mo and W elemental electron core energy levels in the alloy core–shell sample (Figure S17c,d). The Mo/W molar ratio of the precursor solution influenced the Mo_*x*_W_1–*x*_S_2_ alloy core–shell dimension and alloy compositions, as illustrated in Figure 5h,i. For example, a 2:1 Mo/W ratio yielded a MoS_2_ core with a WS_2_-like alloy shell (i.e., an alloy closer in Raman signature to the signature of pure WS_2_), whereas the decrease to a 1:8 Mo/W ratio resulted in a MoS_2_-like core with a WS_2_ shell. The core–shell structure evidently results from differences in vapor-phase or on-surface transport kinetics for the W and Mo precursors.^50,51^

In contrast, two sequential Hy-MOCVD growths of W followed by Mo precursors resulted in WS_2_–MoS_2_ in-plane heterostructures (Figure 5j,k). Raman spectra collected from two sides of the WS_2_–MoS_2_ heterostructure show distinct MoS_2_ and WS_2_ peaks without significant alloying (Figure 5l), and a Raman spectrum line scan shows a distinct interface between WS_2_ and MoS_2_ (Figure 5m). Multilayer MoS_2_ nucleation also occurred on top of WS_2_ and at the interface of the heterostructure. This shows the capabilities of Hy-MOCVD for growing WS_2_–MoS_2_ heterostructures with different layer numbers and vertical stacking.

## Conclusions

In summary, we have demonstrated that Hy-MOCVD provides an effective strategy for rapidly synthesizing TMDC monolayers with diverse transition metal dopants, alloy elements, and heterostructures, offering a versatile platform for exploring synthesis to realize enhanced and tailored electronic, optical, and magnetic properties in TMDC monolayers and heterostructures.

## Experimental Methods

### Material Growth and Transfer

Hy-MOCVD commenced with the preparation of an initial aqueous solution comprising transition metal precursors and promoters. In the case of pure WS_2_ growth, 0.6 g of AMT and 0.05–0.1 g of KOH were dissolved in 30 mL of DI water. For V-doped WS_2_ growth, around 90 mg of NaVO_3_ was introduced into the 30 mL AMT + KOH solution to achieve 24% V/(V + W) atom mole ratio in the solution. Ultrasonication was employed to facilitate the dissolution of NaVO_3_. NaVO_3_ was not fully dissolved, and the cloudy solution was used for growing a 24% V-WS_2_ sample. The cloudy solution was diluted multiple times to get 3 and 0.3% V/(V + W) atom mole ratio solutions. In these low V/(V + W) ratio solutions, NaVO_3_ appeared to be fully dissolved. For Re-doped WS_2_ growth, NH_4_ReO_4_ was used as a Re source. For Mo_*x*_W_1–*x*_S_2_ alloy growth, AMT + KOH (0.6 g + 0.05 g in 30 mL DI water) and ammonium molybdate + KOH (0.43 + 0.2 g in 30 mL DI water) solutions were made separately and mixed with different volume ratios from 2/1 to 1/8. For the growth of WS_2_–MoS_2_ heterostructures, two-step dip-coating Hy-MOCVD was used to grow WS_2_ and MoS_2_ sequentially. In the dip-coating path of Hy-MOCVD, the aqueous solution was dip-coated onto one or all edges of ozone-treated sapphire substrates, followed by N_2_ blow drying. For the dip-coating Hy-MOCVD growth on a 2 inch *c*-plane sapphire wafer, in addition to coating the wafer edge, two initial solution coated W foil strips were placed on the top of the wafer, forming a cross and sitting at its center. In the spin-coating path of Hy-MOCVD, 0.25 mL of 10–16 times diluted initial solution was spin-coated onto ozone-treated sapphire and Si/SiO_2_ substrates at 1000 rpm for 1 min. When growing on Si/SiO_2_ and sapphire/Au, no ozone was applied before dip-coating. For the growth of WS_2_ on *a*-plane sapphire (Hefei Crystal Technical Material Co., Ltd., *a*-plane off *c*-plane 1.0 ± 0.1°), the wafer was annealed in a muffle furnace at 1200 °C for 12 h in an ambient air environment. The *c*-plane sapphire wafers (Valley Design Corp., 28362-1) used in this paper were not annealed. The solution-coated substrates were baked on a hot plate at 80 °C for 1 min and quickly loaded into a MOCVD tube furnace. The tube was evacuated to <0.5 Torr and filled with a flowing mixture of 1600 sccm Ar and 10 sccm H_2_. The furnace temperature was ramped to 725–775 °C over 30 min. Changes to the growth temperature will modify the active concentration of the transition metal and growth promoter species on the substrate surface, and therefore, the composition of the precursor solution may need to be separately optimized for large changes in growth temperature. Subsequently, the H_2_ flow was adjusted to 1 sccm, and 0.05–0.12 sccm of DES was introduced into the tube furnace. The substrates underwent annealing in this environment for 2–6 h to complete growth. Postgrowth, the DES flow was reduced to 0.025–0.1 sccm, and the furnace heating was discontinued. DES flow was closed when the furnace naturally cooled to 300 °C, and substrates were unloaded at room temperature.

WS_2_ grown on sapphire substrates was transferred onto Si/SiO_2_ substrates using a poly(methyl methacrylate) (PMMA)-assisted transfer method. The samples were spin-coated with PMMA and dried on a hot plate at 100 °C for 3 min. WS_2_/PMMA was delaminated from the sapphire substrate by gradually dipping the substrate into DI water (the substrate was in an upward-facing position and angled at 30–60° relative to the water surface) and transferred onto the target substrate with SiO_2_ (100 nm) on Si, followed by drying on a hot plate at 100 °C for 5 min. The PMMA layer was removed by soaking it in acetone at 60 °C for 15 min.

### Device Fabrication and Analysis

For the transferred devices shown in Figure 4c, monolayer WS_2_ was grown on sapphire with dip-coating Hy-MOCVD and transferred off by using a PMMA-based transfer (as described above) onto 100 nm SiO_2_ on Si. For the devices fabricated on the Si/SiO_2_ growth substrate, shown in Figure 4d, dip-coating Hy-MOCVD monolayer WS_2_ was directly grown on SiO_2_ (100 nm) on p^++^ Si (≤0.005 Ω·cm) that also served as the back-gate. Alignment marks were first patterned on the direct-grown sample, such that discrete WS_2_ crystals could be identified. Devices were made on single crystalline WS_2_ triangles to avoid the existence of grain boundaries in the device channels. The measured devices were sampled randomly from within a 5 × 5 mm^2^ region on each chip. Electron-beam lithography was employed for each lithography step. Large probing pads (SiO_2_/Ti/Pt 10/2/20 nm) were first patterned and deposited by electron-beam evaporation via lift-off. SiO_2_ was used in the probing pad to limit the pad-to-substrate leakage. XeF_2_ was used for channel definition, and the contact region was patterned for lift-off. 15/30 nm Ni/Au contacts were electron-beam evaporated at ∼10^–8^ Torr, and a rate of 0.5 Å/s. 20/35 nm Ni/Au contacts were deposited for the nontransferred devices. The fabricated transistors were measured in a Janis ST-100 probe station at ∼10^–4^ Torr under vacuum using a Keithley 4200 semiconductor parameter analyzer.

For the undoped and the V-doped WS_2_ devices shown in Figure 5e, the starting WS_2_ and V-WS_2_ were grown on 100 nm SiO_2_ on Si with spin-coating Hy-MOCVD. Alignment marks were patterned to identify monolayer regions on both samples. Metal pads and channels were defined by e-beam lithography, as described above. For both the doped and undoped WS_2_, Ru/Au (5/50 nm) were deposited via e-beam evaporation to investigate potential p-type transport from V-doped WS_2_, based on previous reports of good p-type performance from Ru contacts.^42^ The devices were measured under vacuum as described above.

Threshold voltage was extracted at a constant current of 10 nA/μm.^52^ The field-effect mobility μ_e_ = max(*g*_m_)/[*C*_ox_*V*_DS_(*W*_ch_/*L*_ch_)], was estimated using the maximum transconductance of forward and backward *V*_GS_ sweeps, *g*_m_ = d*I*_D_/d*V*_GS_, and the gate insulator capacitance per unit area is *C*_ox_ = ε_0_κ_ox_/*t*_ox_. The SiO_2_ gate oxide thickness *t*_ox_ = 100 nm, the oxide relative permittivity κ_ox_ = 3.9, ε_0_ is the vacuum permittivity, and *V*_DS_ = 1 V. *W*_ch_ and *L*_ch_ are channel width and length, respectively. The designed *W*_ch_ was 2.0 μm. The final *W*_ch_ was measured via SEM to be 1.6 μm for FETs of transferred WS_2_ and 2.0 μm for FETs of as-grown WS_2_. The final *L*_ch_ in the FETs of transferred WS_2_ were measured via SEM to be 72, 175, 261, 461, 650, and 973 nm, corresponding to the designed *L*_ch_ values of 100, 200, 300, 500, 700, and 1000 nm, respectively. The final *L*_ch_ in the FETs of as-grown WS_2_ were measured via SEM to be 173, 275, 477, 681, and 993, corresponding to the designed *L*_ch_ values of 200, 300, 500, 700, and 1000 nm, respectively. The mobilities of the FETs were corrected with these measured *W*_ch_ and *L*_ch_ values.

### Material Characterizations

AFM imaging was conducted utilizing a Bruker ICON AFM using the ScanAsyst topography imaging mode with a NSC19/Al-BS tip. Raman and PL spectra were acquired at room temperature with 532 nm laser excitation using a HORIBA Scientific LabRAM HR Evolution confocal microscope. Optical microscope imaging was performed using an Olympus BX-51 microscope in epi-reflection geometry. The optical microscope contrast for images in Figures 2c,g and 3b–e,g–i,k were enhanced in the following way: after acquisition, we converted the color images to grayscale and increased the contrast and brightness to improve visibility of the WS_2_ on the transparent sapphire wafer. SHG was performed using a femtosecond laser (NKT Origami Onefive 10, 1030 nm, <200 fs) at room temperature. A 40× objective lens was used to excite the sample with an average power of 5–10 mW, and the signal was collected in reflective geometry by an EMCCD (Andor iXon Ultra) with an integration time of 100 ms at each polarization angle. XPS was performed using PHI VersaProbe 3.

## Supporting Information Available

Schematic of Hy-MOCVD setup, high magnification AFM of spin-coating grown WS_2_, PL peak fitting results, XPS of potassium and carbon element, growth results over 17 months, optical images and Raman of MoS_2_ and WSe_2_, continuous WS_2_ monolayer domain size extraction, AFM of WS_2_ grown on *a*-plane sapphire, SHG characterizations of WS_2_ ribbons grown on *a*-plane sapphire, wide range Raman spectra of WS_2_ grown on sapphire and SiO_2_, *L*_ch_ vs *I*_D_, mobility, hysteresis, and typical *I*_D_ vs *V*_GS_ hysteresis, PL, and Raman comparison between as-grown and transferred WS_2_, and Raman 2LA + E′ peak intensity vs nominal doping concentration of V-doped WS_2_, XPS of V-doped WS_2_, PL of V-doped and Re-doped WS_2_, XPS of alloy, tables of the comparison of growth metric, MOCVD growth parameters, and FET performance (PDF)
