# Supplementary material for: Chemically Tailored Growth of 2D Semiconductors via Hybrid Metal–Organic Chemical Vapor Deposition
Source: ACS Nano. 2024 Sep 4;18(37):25414–24. doi: 10.1021/acsnano.4c02164 (PMC11412230; doi:10.1021/acsnano.4c02164)
Supplement: Supplementary file 1 — nn4c02164_si_001.pdf [file nn4c02164_si_001.pdf]

Supporting Information for "Chemically Tailored Growth of 2D Semiconductors *via* Hybrid Metal-Organic Chemical Vapor Deposition"

*Zhepeng Zhang[1,#], Lauren Hoang[2,#], Marisa Hocking[1], Zhenghan Peng[1], Jenny Hu[3], Gregory Zaborski Jr.[1], Pooja Reddy[1], Johnny Dollard[1], David Goldhaber-Gordon[4,5], Tony F. Heinz[3,5,6], Eric Pop[1,2,7], Andrew J. Mannix[1,5]**

[1]Department of Materials Science & Engineering, Stanford University, Stanford, CA 94305, USA
[2]Department of Electrical Engineering, Stanford University, Stanford, CA 94305, USA
[3]Department of Applied Physics, Stanford University, Stanford, CA 94305, USA
[4]Department of Physics, Stanford University, Stanford, CA 94305, USA
[5]Stanford Institute for Materials and Energy Sciences, SLAC National Accelerator Laboratory, Menlo Park, CA 94025, USA
[6]Department of Photon Sciences, Stanford University, Stanford, CA 94305, USA
[7]Precourt Institute for Energy, Stanford University, Stanford, CA 94305, USA

*Corresponding author: A.J.M., ajmannix@stanford.edu

#These authors contributed equally to this work (Z.Z., L.H.).

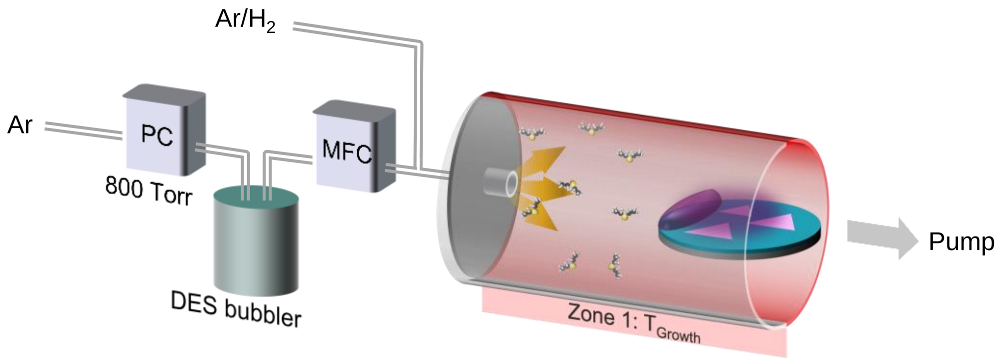

Figure S1. Schematic of Hy-MOCVD setup. PC and MFC denote pressure controller and mass flow controller, respectively.

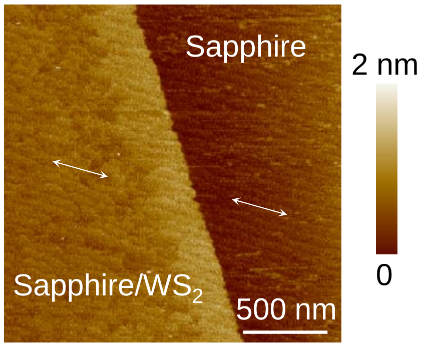

Figure S2. High magnification AFM image of spin-coating Hy-MOCVD WS_2 on *c*-plane sapphire substrate. Arrows highlight the directions of steps visible in sapphire/WS_2 and sapphire regions.

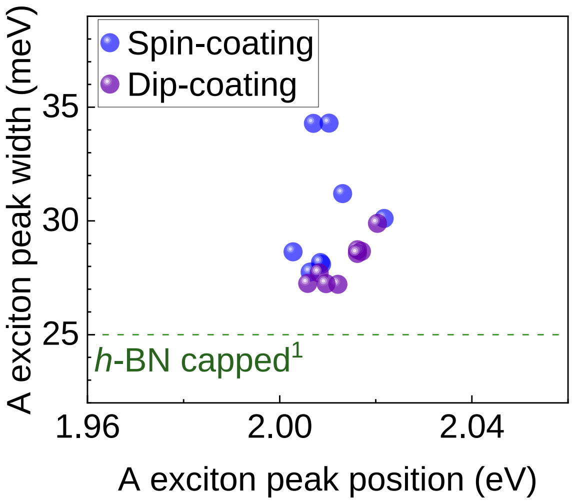

Figure S3. Fitted A exciton PL peak position versus peak width of as-grown spin-coating and dip-coating Hy-MOCVD WS_2. The Hy-MOCVD WS_2 monolayers present narrow A peak width distribution close to the *h*-BN capped WS_2.[1]

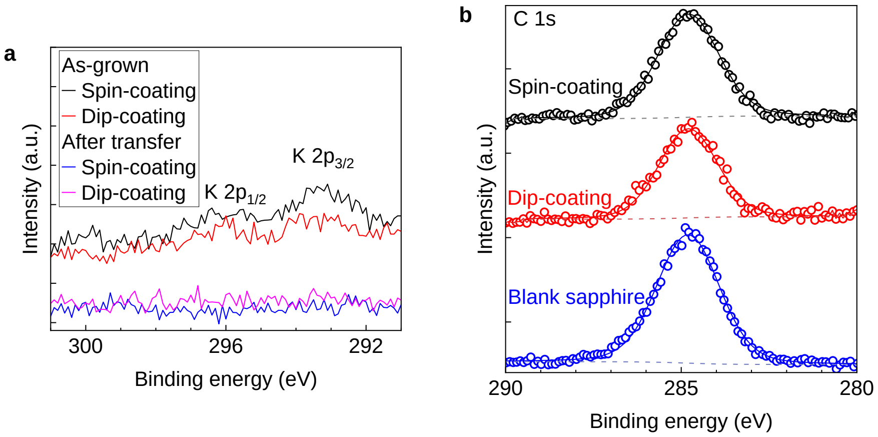

Figure S4. (a) High-resolution XPS spectra for the K 2p core level on Hy-MOCVD, before and after transfer, for both spin-coating and dip-coating precursor delivery. (b) C 1s core level spectra comparing a bare sapphire wafer with Hy-MOCVD films grown from dip-coating and spin-coating precursor delivery, indicating the absence of additional carbon contamination from the Hy-MOCVD process (data points shown with accompanying Voight profile curve fits).

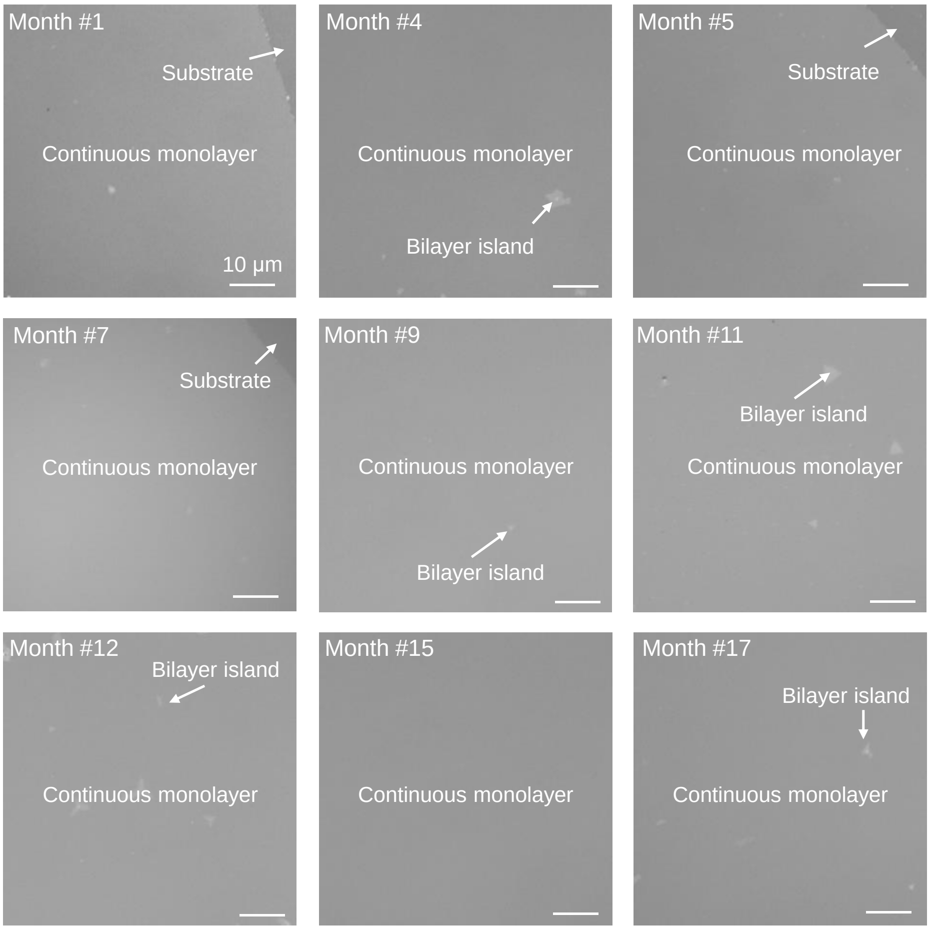

Figure S5. Optical images of dip-coating Hy-MOCVD grown monolayer WS_2 films on *c*-plane sapphire in 17 months. Scratch exposed substrate areas and bilayer islands on monolayer WS_2 are indicated. The parameters for these growths are identical. AMT + KOH (0.6 g + 0.05 g in 30 ml DI water) solution, 0.05 sccm DES, 1600 sccm Ar, 1 sccm H_2 and 775 °C growth temperature were used for the growth.

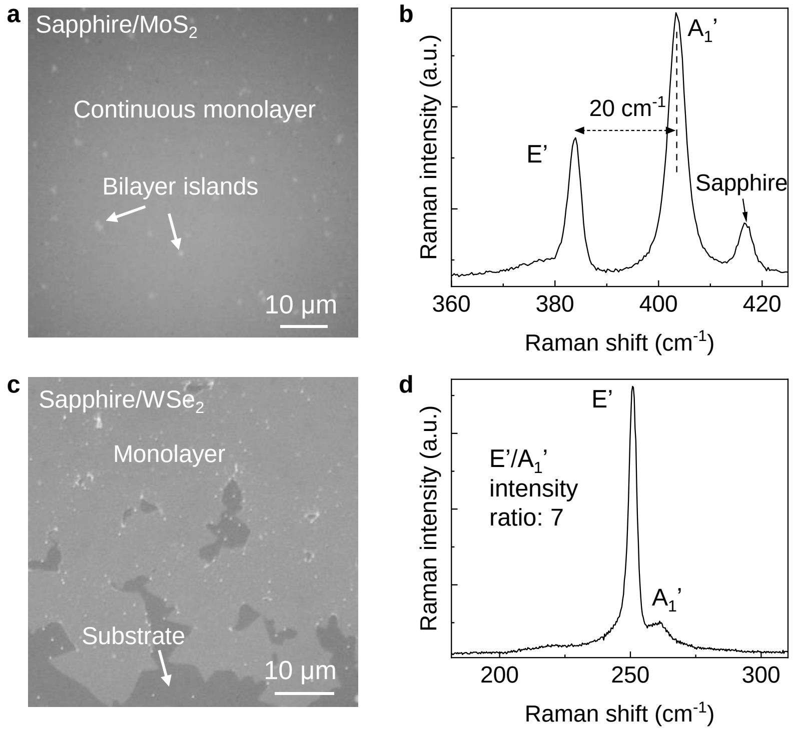

Figure S6. Optical images (a,c) and Raman spectra (b,d) of dip-coating Hy-MOCVD grown monolayer MoS_2 and WSe_2 films on *c*-plane sapphire substrates. The MoS_2 E' to A_1' peak distance of 20 cm^{-1} and the WSe_2 E'/A_1' peak intensity ratio of 7 indicate the predominantly monolayer character of Hy-MOCVD grown MoS_2 and WSe_2, respectively. Ammonium molybdate tetrahydrate and diisopropylselenium were used as Mo- and Se-sources for the Hy-MOCVD growth of MoS_2 and WSe_2, respectively.

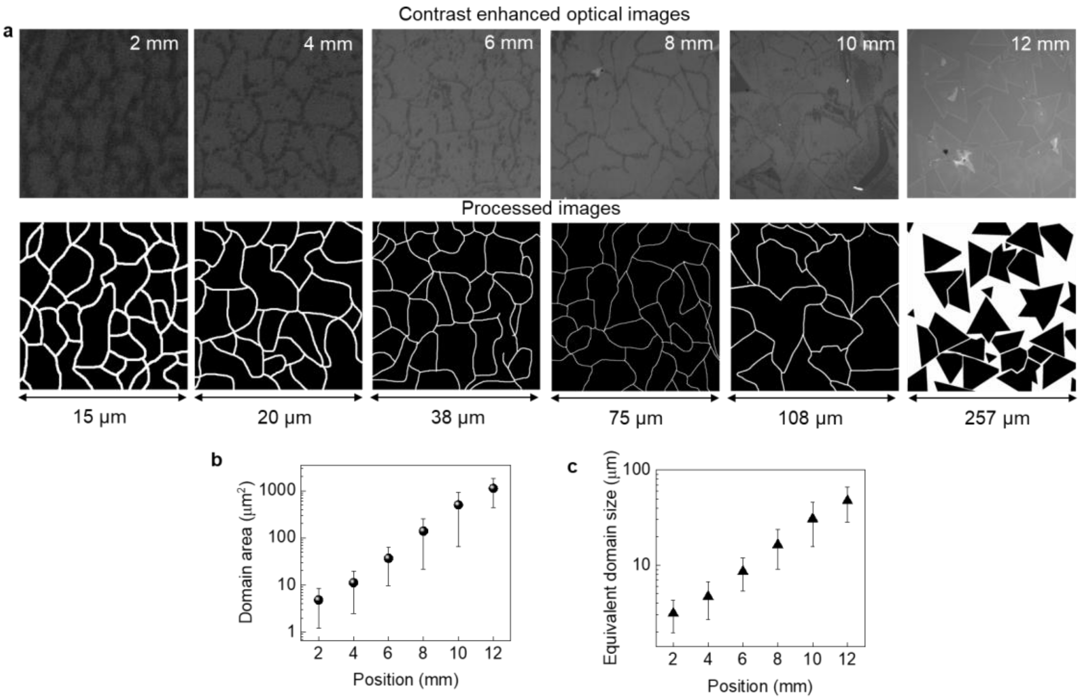

Figure S7. The domain size extraction of dip-coating Hy-MOCVD WS_2 continuous monolayer film on sapphire substrate. **(a)** Contrast enhanced optical images (top) and processed images of 7 min ozone etched WS_2 continuous monolayer films and domains on different positions 2-12 mm away from the dip-coating edge in dip-coating Hy-MOCVD. **(b)** Extracted domain areas versus position plot. **(c)** Equivalent domain size versus position plot. Particle analysis function in Image J was used to extract domain areas from processed images. The domain shape was assumed to be equilateral triangle for converting domain area to equivalent domain size (edge length of the equilateral triangle).

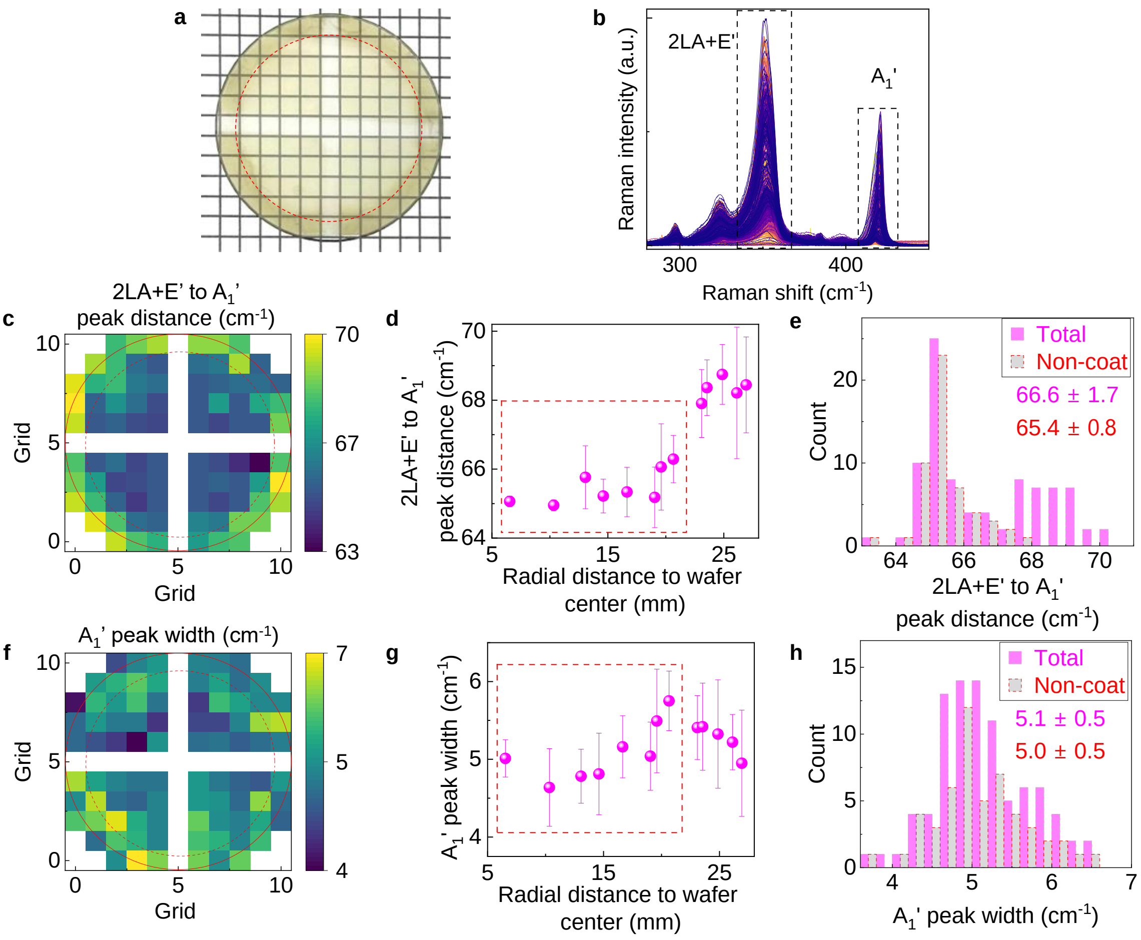

Figure S8. Raman map of Hy-MOCVD WS_2 on 2" sapphire wafer. (a) Photo of a 2" wafer on a grid paper. Red dashed circle highlights the WS_2 growth area during the Hy-MOCVD growth. (b) Raman spectra of 3×3 data points over 40×40 μm^2 area within each square of the total 88 squares on the wafer. The squares in the W trip covered area are excluded. The maps of 2LA+E' to A_1' peak distance (c) and A_1' peak width (f). The pixel intensities are the average values of the 9 spectra collected in each square. The red line and red dash line highlight the area of 2" wafer and non-dip-coated area. (d) and (g) show the average intensity and peak widths along the radius direction in (c) and (f), respectively. Note that the radial distance is calculated with respect to the grid coordinate, and therefore can exceed the 1" (25.4 mm) radius expected for a 2" (50.8 mm) diameter wafer. The error bars are standard deviations. (e) and (h) are histograms of the intensities in (c) and (f), respectively. Average values and standard deviations of all the pixels and the pixels in non-coated area are indicated. The peak positions and widths were extracted using Horiba LabSpec 6.

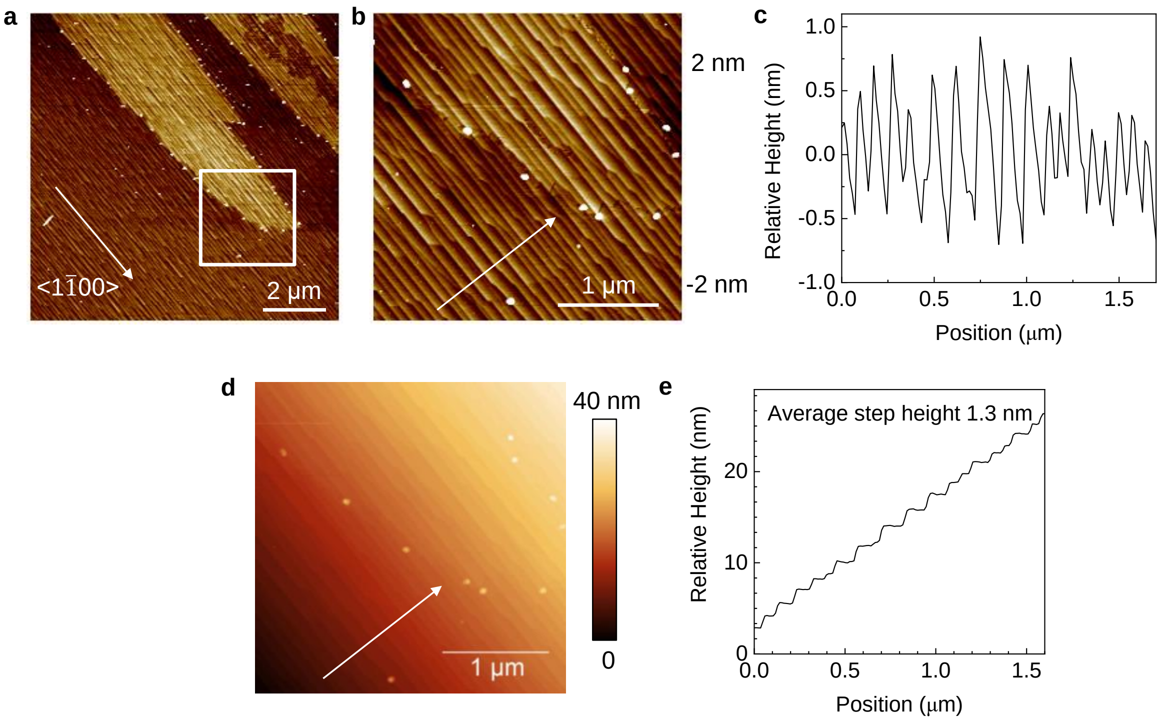

Figure S9. **(a)** AFM image of WS_2 ribbon grown on annealed *a*-plane sapphire wafer with terraces. **(b)** Zoomed in AFM image from the square in **(a)**. **(c)** Height line profile of sapphire wafer terraces along the white arrow in **(b)**. **(d)** Zoomed in AFM image from the square in **(a)**. **(e)** Height line profile of sapphire wafer terraces along the white arrow in **(d)**. **(a)** and **(b)** are flattened with NanoScope Analysis. **(d)** is flattened with Gwyddion.

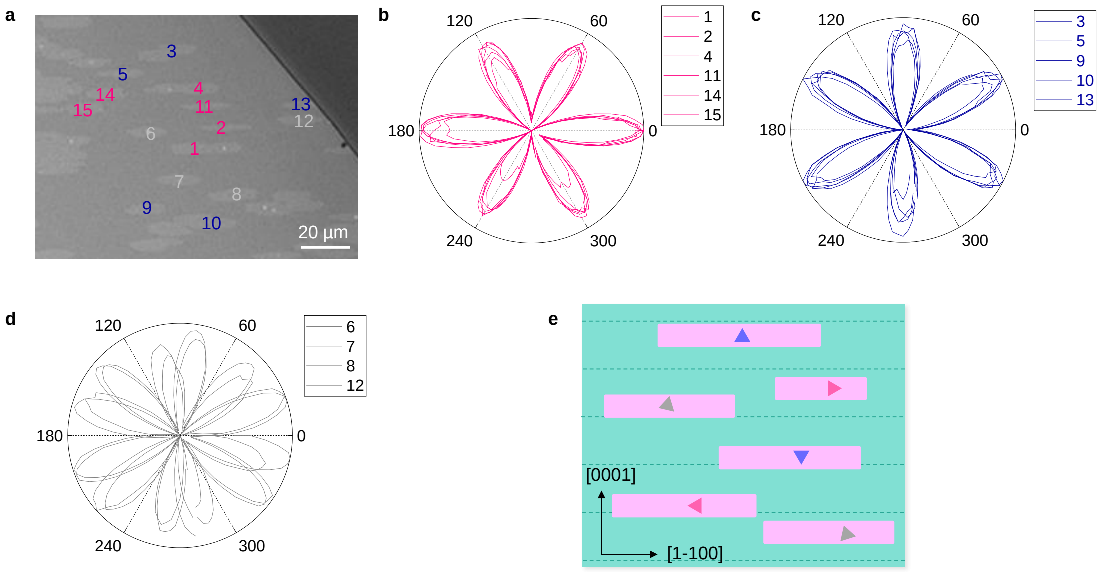

Figure S10. (a) Contrast-enhanced optical image of Hy-MOCVD grown WS_2 ribbons on annealed *a*-plane sapphire. **(b-d)** Polarization-resolved SHG of the ribbons highlighted in **(a)**. The SHG signals are normalized based on their maximum intensities. The armchair orientations of the WS_2 lattice within the ribbons are classified into three distinct groups: parallel (**b**), perpendicular (**c**), and randomly orientated (**d**) with respect to the long axis of the ribbon. **(e)** Schematic of WS_2 ribbons grown on an *a*-plane sapphire substrate featuring consistent ribbon orientation but varying lattice orientations. The corners of the triangles denote the armchair directions of the WS_2 lattice within the ribbon. Dashed lines indicate the terrace edge directions on the annealed *a*-plane sapphire.

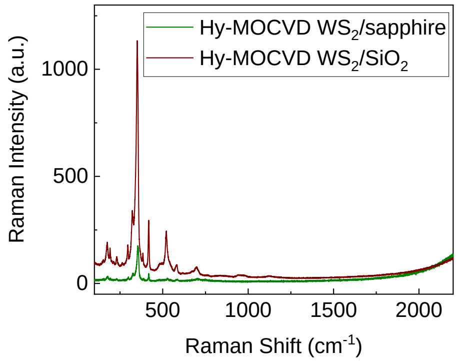

Figure S11. Raman spectra of as-grown Hy-MOCVD WS_2 on sapphire and SiO_2 showing no amorphous carbon signal.

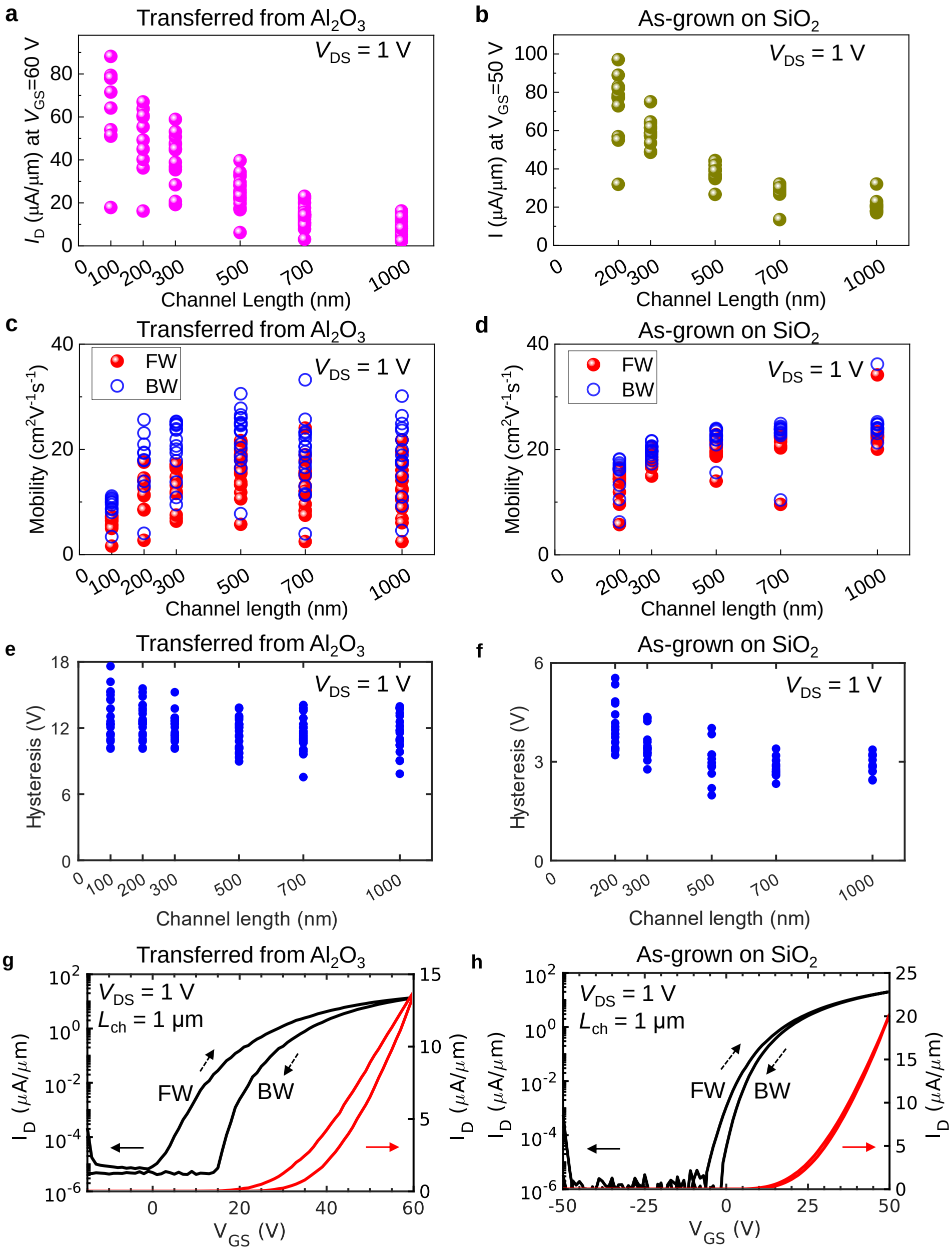

Figure S12. (a,b) On-state current (V_{DS} = 1 V) vs. channel length of FETs for transferred and as-grown Hy-MOCVD WS_2, respectively. **(c,d)** Estimated field-effect mobility vs. channel length of FETs for transferred and as-grown Hy-MOCVD WS_2, respectively. **(e,f)** Hysteresis vs. channel length of FETs for transferred and as-grown

Hy-MOCVD WS_2, respectively. **(g,h)** Repetitive I_D vs V_{GS} curves of FETs for transferred and as-grown Hy-MOCVD WS_2 showing typical clockwise hysteresis, respectively. FW and BW stand for forward and backward I_D vs V_{GS} curves, respectively.

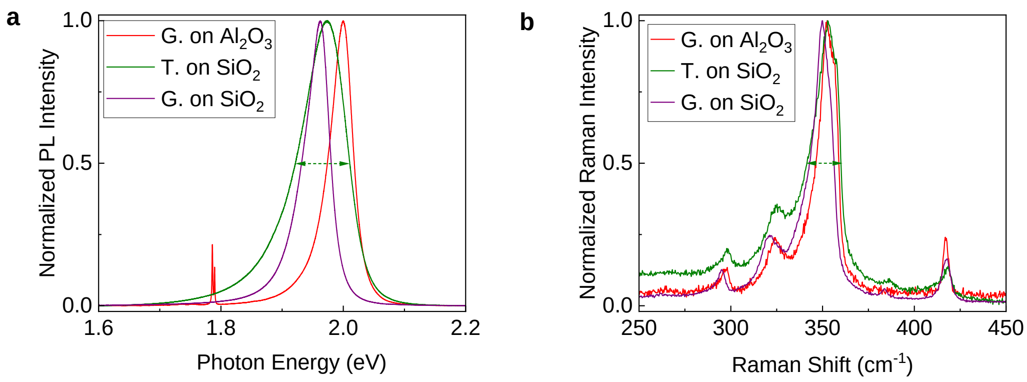

Figure S13. (a,b) PL and Raman spectrum comparison between as-grown Hy-MOCVD WS_2 on sapphire, transferred Hy-MOCVD WS_2 on from sapphire onto Si/SiO_2, and as-grown Hy-MOCVD WS_2 on Si/SiO_2. "G. on Al_2O_3" refers to "As-grown WS_2 on sapphire". "T. on SiO_2" is defined as "Transferred WS_2 from sapphire to SiO_2". "G. on SiO_2" is denoted as "As-grown WS_2 on SiO_2".

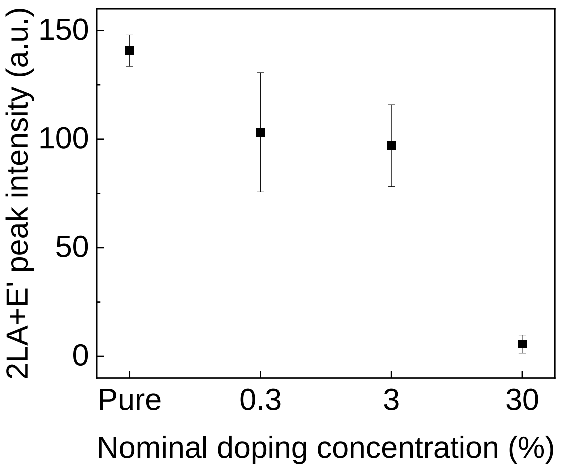

Figure S14. Raman 2LA+E' peak intensity versus nominal doping concentration plot of Hy-MOCVD grown V-doped WS_2.

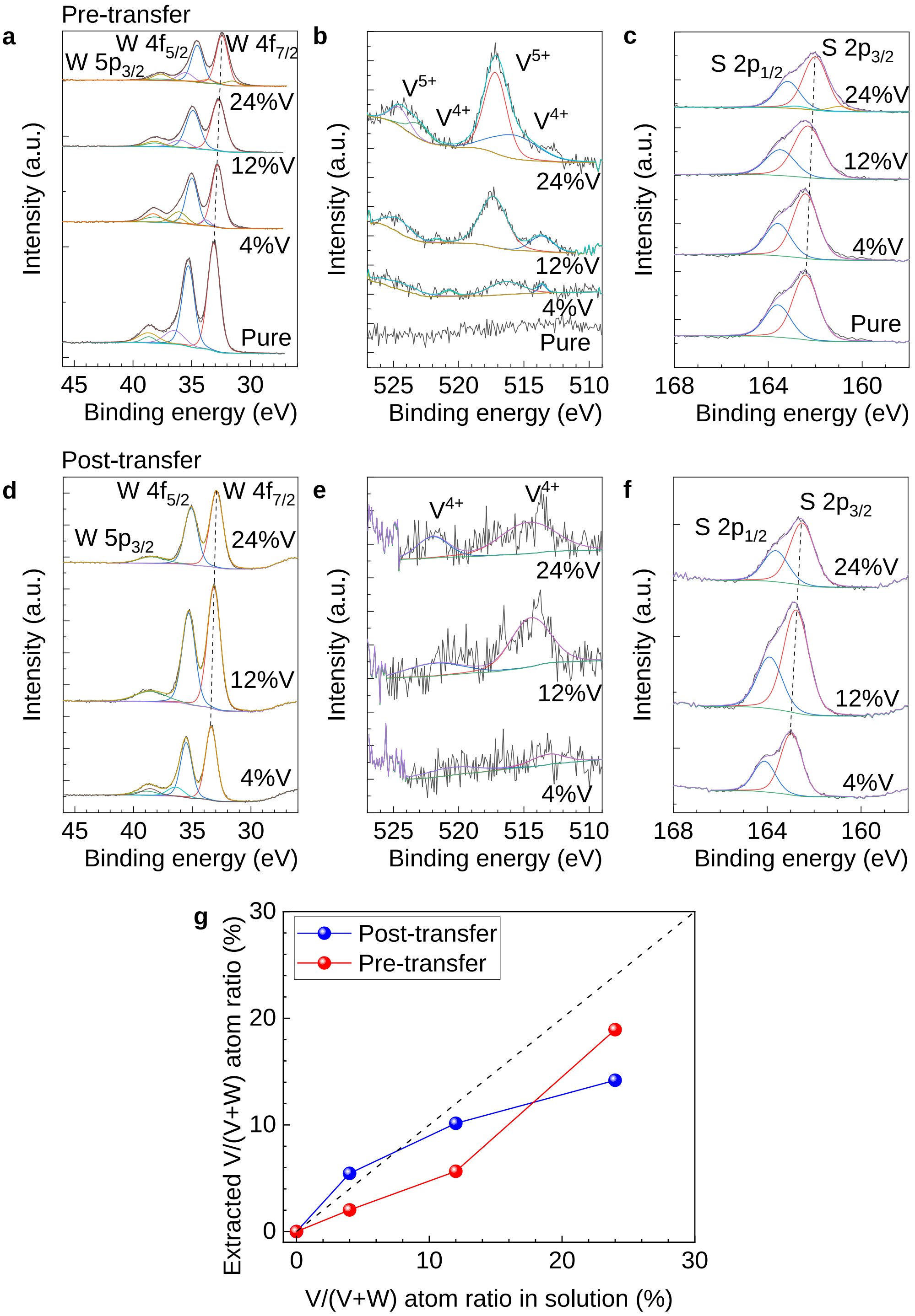

Figure S15. XPS characterization of Hy-MOCVD V-doped WS_2 before and after transfer. (a-c) High resolution XPS spectra of W 4f, V 2p and S 2p core levels for Hy-MOCVD V-doped WS_2 before transfer. (d-f) High resolution XPS spectra of W 4f, V 2p and S 2p core levels for Hy-MOCVD V-doped WS_2 after transfer. Black dashed lines in (a,c,d,f) connect the peak maxima for the undoped and 24%V samples to highlight the decreasing binding energy trend in W 4f and S 2p core levels. (g) Nominal V/(V+W) atom ratio in the precursor solution versus the ratio measured with XPS. The

measured XPS atomic ratio is calculated using the area of the V^{4+} component peak, because this is the expected chemical state for V substitution of W^{4+} in the WS_2 lattice. The samples were grown on *c*-plane sapphire for the pre-transfer measurements and transferred onto an Si/SiO_2 (100 nm) substrate for the post-transfer measurements. XPS peak fittings were completed on CasaXPS,[2] using the corrected relative sensitivity factors from the MultiPak XPS data processing software for the PHI VersaProbe 3 system. The residual standard deviations for V 2p fittings are in the range of 0.84~1.17. The W oxide states and V^{5+} states in pre-transfer samples can come from incomplete sulfurization of the W- and V- precursors. Their intensities are greatly reduced in the post-transfer samples, perhaps due to their water solubility or adhesion to the growth substrate.

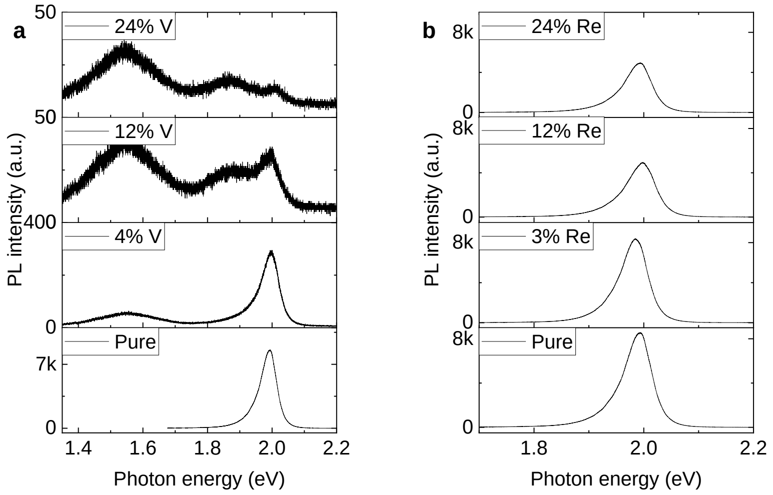

Figure S16. Series of photoluminescence spectra for Hy-MOCVD-grown V-doped (a) and Re-doped (b) WS_2 at room temperature. The samples were grown on sapphire and transferred to SiO_2 substrates. The dopant concentration values are given as the nominal dopant concentration in the solution and were deposited *via* spin coating.

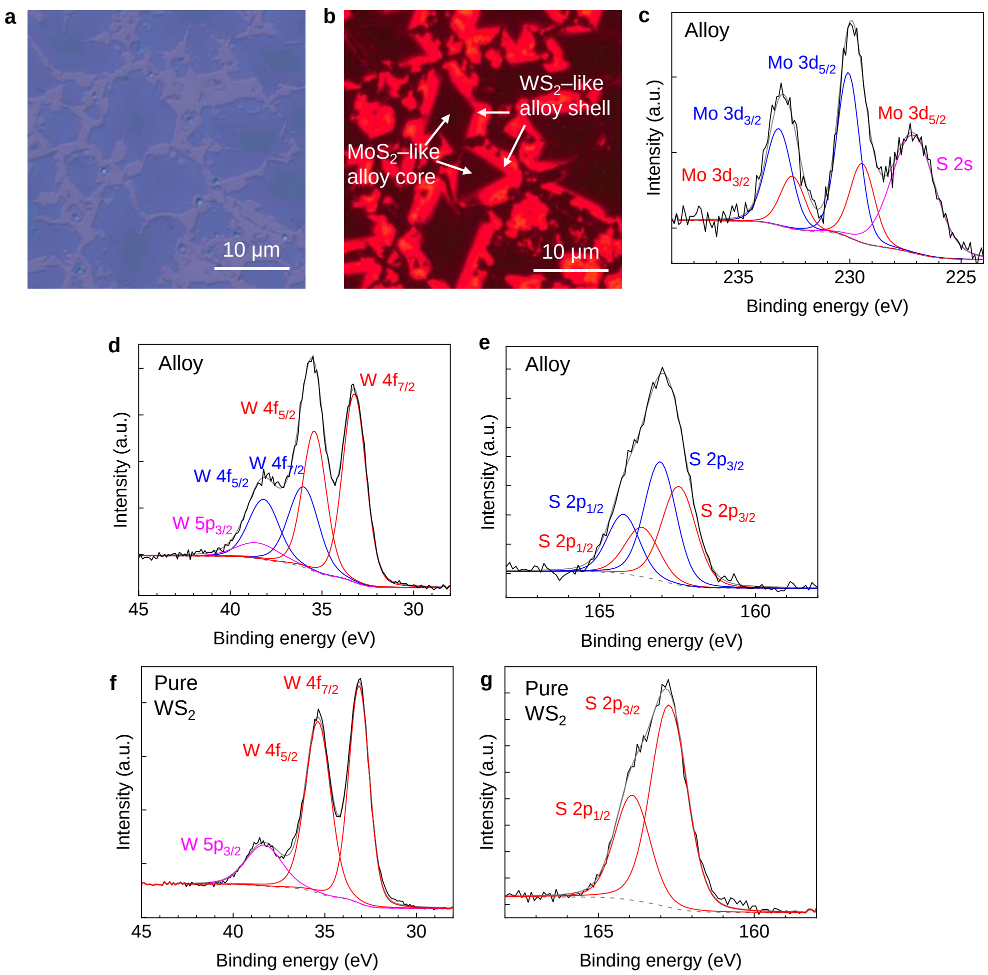

Figure S17. Characterization of $Mo_xW_{1-x}S_2$ alloy samples. (a) Optical microscope image (aperture stop applied) of transferred $Mo_xW_{1-x}S_2$ alloy grown with Mo/W mole ratio of ½ in the initial solution. (b) The fluorescence image of the transferred $Mo_xW_{1-x}S_2$ alloy in the same area of (a). (c-e) High-resolution XPS spectra of Mo 3d, W 4f and S 2p of as-grown $Mo_xW_{1-x}S_2$ alloy sample. (f-g) High-resolution XPS spectra of W 4f and S 2p of as-grown pure WS_2 sample. XPS peak fittings were completed on CasaXPS.[2]

Table S1. Summary of the metrics of SS-CVD, Hy-MOCVD and MOCVD.

**Metrics**	**SS-CVD**	**Hy-MOCVD**	**MOCVD**
Domain size	• *Non-epitaxial:*<br>< 100 μm for isolated domains of MoS_2[3] and WS_2.[4]<br>2-5 μm for continuous film of MoS_2.[5,6]<br>100s μm for isolated domains of MoS_2[7] on sapphire (O_2 assisted) and WS_2 grown on Au foil[8]<br>• *Unidirectional epitaxy:*<br>10-20 μm for isolated domains of MoS_2[9,10] and WS_2.[11] (closed epitaxial film)	3-30 μm for continuous film (**Figure S7**);<br>~60 μm for isolated domains (**Figure 3i**)	• *No growth promoter:*<br>100s nm for continuous film.[12,13]<br>20 μm for isolated domians.[14]<br>• *Using growth promoter:*<br>100s nm for continuous film.[15–17]<br>1 μm ~ 30 μm for continuous film.[18,19]<br>60 μm for isolated domains.[20]<br>• *Using water etchant:*<br>100s nm ~ 15 μm for isolated domains.[21]<br>• *Using reverse flow:*<br>120 μm for isolated domains.[22]<br>100s nm for continuous film.[23]
Growth time	~0.5 hours	2-6 hours (**Figure 2** and **3**)	10 minutes - 30 hours (**Table S2**)
Repeatability	• *Conventional source delivery:*<br>Small variations of source amount and position modify the growth result dramatically.<br>• *Special oxide delivery:*<br>Improved consistency in oxygen assisted MoS_2 growth.[6,24]	Consistency between the growth over 17 months (**Figure S5**)	Excellent repeatability (precisely controlled metal and chalcogen source deliveries).
Composition and doping control	Potential for large metal/chalcogen ratio variation for standard oxide delivery, MoS_2.[25]	Precisely controlled organochalcogen flow rate. Easy transition metal engineering for doping and alloying (**Figure 5**)	Each new precursor or dopant source requires either adding a new vapor-phase delivery line to the MOCVD system or swapping for an existing source, with potential for contamination and memory effects.

Uniformity	• *Conventional oxide delivery:*<br>Shape and coverage vary in hundred micrometer length, MoS_2.[25]<br>• *With special oxide delivery:*<br>2-4" wafer continuous film, MoS_2, oxygen assisted.[6,24]<br>12" wafer continuous film, MoS_2.[5]<br>• *With salt promoter:*<br>2" wafer continuous film, WS_2.[11]	~80% of the growth area on 2" wafer is uniformly monolayer and usable for device fabrication (**Figure 3j** and **Figure S8**)	Excellent wafer scale uniformity[15,23,26]

Table S2. Comparison of MOCVD growth parameters in literature.

**MX_2**	**Heating (CW/HW)**	**T_{Gr} (°C)**	**t_{Gr}**	**Promoter**	**Precursors**	**Flow rate (sccm)**	**Special design**	**Ref.**
MoS_2, WS_2	CW	1000	10-18 min	-	$Mo(CO)_6$, $W(CO)_6$, H_2S	10^{-4}, 400	Nucleation + Ripening	26
WS_2	CW	1000	10-45 min	-	$W(CO)_6$, H_2S	10^{-4}, 400	Nucleation + Ripening	13
WS_2	HW	850	30-40 min	-	$W(CO)_6$, DTBS	10^{-7}, 10^{-4}	Water etching-growth	21
MoS_2	HW	320	2-14 h	-	$W(CO)_6$, $(C_2H_5)_2S$	0.1, 2	Backward flow	22
MoS_2	HW	300	<1 h	Spin NaCl	$Mo(CO)_6$, $(C_2H_5)_2S$	120, 80	Backward flow	23
MoS_2	HW	150	31 h	-	$Mo(CO)_6+C_2H_6S$	0.6	Mix Mo and S sources	12
MoS_2, WS_2	HW	550	26 h	NaCl powder	$Mo(CO)_6$, $W(CO)_6$, $(C_2H_5)_2S$	0.01, 0.01, 0.4	-	15
$MoSe_2$, WSe_2	CW	800	1.5 h	-	$Mo(CO)_6$, $W(CO)_6$, H_2Se	10^{-4}, 10^{-4}, 7	Nucleation + Ripening	14
MoS_2	CW	250	8 h	NaCl powder	$Mo(CO)_6$, H_2S	-	-	16

MoS_2	HW	700	1 h	-	$Mo(CO)_6$, $(C_2H_5)_2S$	0.02, 0.3-13.2	-	17
MoS_2	HW	800-1050	30 min	KI/NaCl powder	$Mo(CO)_6$, H_2S	10^{-4}, S-rich	-	20
MoS_2, WS_2, $MoSe_2$, WSe_2	HW	600	4 h	$C_3H_5NaO_2$	$Mo(CO)_6$, $W(CO)_6$, $(C_2H_5)_2S$, $(CH_3)_2Se$	1.2, 3.5, 0.4, 0.5	Gas phase Na-promoter	18
MoS_2	HW	900	1 h	NaCl powder	$Mo(CO)_6$, $(C_2H_5)_2S$	2, 4	-	19

Abbreviations: MX_2 stands for the TMDC composition of M as the transition metal (Mo, W), and X as the chalcogen (S, Se); CW and HW refer to cold wall and hot wall, respectively; T_{Gr} refers to growth temperature; t_{Gr} denotes growth time; Ref. stands for Reference.

Table S3. Performance comparison between Hy-MOCVD WS_2 FET devices and previous reports.

**Ref.**	**Method**	**Contacts**	**Gate Dielectric**	**Electron mobility μ_e ($cm^2V^{-1}s^{-1}$)**	**Max I_D (µA/µm) at V_{DS}= 1 V**	**L_{ch} (nm)**	**Current on/off ratio**
This work	Hy-MOCVD	Ni/Au	100 nm SiO_2	35 (max)	88 (max)	100-1000	10^8(max)
27	CVD	Au	100 nm SiO_2	-	10	600	10^7
28	CVD	Bi	100 nm SiN_x	18	300	100	-
29	CVD	Bi/Au	300 nm SiO_2	21	100	150	10^7
30	CVD	Bi/Au	100 nm SiNx	200	400	100	10^8
31	CVD	Bi/Ti/Au	16/30 nm SiO_2/Al_2O_3 + 31nm Al_2O_3 DG	15.5	46	320	10^{10}
32	CVD	Sb/Au or Bi/Au	100 nm SiN_x	30	243	135	10^8
33	CVD	Ni/Au	8 nm BeO	26	325	50	10^8

34	CVD	Ni	2.8 nm HfO_2 + 5.5 nm HfO_2 DG	-	225	140	-
35	CVD	Ni/Au	10 nm HfLaO	-	267	80	-
8	CVD	Ti/Au	290 nm SiO_2	2.00	3.3	3000	10^7
4	CVD		Ionic Liquid	0.46	5×10^{-3}	3000 0	10^2
11	CVD		-	1.60	-	-	-
36	CVD	Cr/Au	300 nm SiO_2	3.00	-	8000	10^8
37	CVD	Cr/Au	SiO_2	13.80	-	-	10^8
38	ALD	Ti/Au	20nm Al_2O_3	6.85	-	-	10^5
26	MOCVD	Ni/Au	50nm Al_2O_3	33	26	100	10^6
13	MOCVD	Ni/Au	50nm Al_2O_3	16	20	100	10^7

Abbreviations: Ref. stands for Reference; DG denotes Dual gate; - refers to no available data; μ_e stands for field-effect electron mobility reported in the reference; I_{on} denotes on-state current; L_{ch} refers to channel length.

REFERENCES

(1) Cadiz, F.; Courtade, E.; Robert, C.; Wang, G.; Shen, Y.; Cai, H.; Taniguchi, T.; Watanabe, K.; Carrere, H.; Lagarde, D.; Manca, M.; Amand, T.; Renucci, P.; Tongay, S.; Marie, X.; Urbaszek, B. Excitonic Linewidth Approaching the Homogeneous Limit in MoS_2-Based van Der Waals Heterostructures. *Phys. Rev. X* **2017**, *7*, 021026.

(2) Fairley, N.; Fernandez, V.; Richard-Plouet, M.; Guillot-Deudon, C.; Walton, J.; Smith, E.; Flahaut, D.; Greiner, M.; Biesinger, M.; Tougaard, S.; Morgan, D.; Baltrusaitis, J. Systematic and Collaborative Approach to Problem Solving Using X-Ray Photoelectron Spectroscopy. *Applied Surface Science Advances* **2021**, *5*, 100112.

(3) Ling, X.; Lee, Y.-H.; Lin, Y.; Fang, W.; Yu, L.; Dresselhaus, M. S.; Kong, J. Role of the Seeding Promoter in MoS_2 Growth by Chemical Vapor Deposition. *Nano Lett.* **2014**, *14*, 464–472.

(4) Zhang, Y.; Zhang, Y.; Ji, Q.; Ju, J.; Yuan, H.; Shi, J.; Gao, T.; Ma, D.; Liu, M.; Chen, Y.; Song, X.; Hwang, H. Y.; Cui, Y.; Liu, Z. Controlled Growth of High-Quality Monolayer WS_2 Layers on Sapphire and Imaging Its Grain Boundary. *ACS Nano* **2013**, *7*, 8963–8971.

(5) Xia, Y.; Chen, X.; Wei, J.; Wang, S.; Chen, S.; Wu, S.; Ji, M.; Sun, Z.; Xu, Z.; Bao, W.; Zhou, P. 12-Inch Growth of Uniform MoS_2 Monolayer for Integrated Circuit Manufacture. *Nat. Mater.* **2023**, *22*, 1324–1331.

(6) Yu, H.; Liao, M.; Zhao, W.; Liu, G.; Zhou, X. J.; Wei, Z.; Xu, X.; Liu, K.; Hu, Z.; Deng, K.; Zhou, S.; Shi, J.-A.; Gu, L.; Shen, C.; Zhang, T.; Du, L.; Xie, L.; Zhu, J.; Chen, W.; Yang, R.; et al. Wafer-Scale Growth and Transfer of Highly-Oriented Monolayer MoS_2 Continuous Films. *ACS Nano* **2017**, *11*, 12001–12007.

(7) Chen, W.; Zhao, J.; Zhang, J.; Gu, L.; Yang, Z.; Li, X.; Yu, H.; Zhu, X.; Yang, R.; Shi, D.; Lin, X.; Guo, J.; Bai, X.; Zhang, G. Oxygen-Assisted Chemical Vapor Deposition Growth of Large Single-Crystal and High-Quality Monolayer MoS_2. *J. Am. Chem. Soc.* **2015**, *137*, 15632–15635.

(8) Gao, Y.; Liu, Z.; Sun, D.-M.; Huang, L.; Ma, L.-P.; Yin, L.-C.; Ma, T.; Zhang, Z.; Ma, X.-L.; Peng, L.-M.; Cheng, H.-M.; Ren, W. Large-Area Synthesis of High-Quality and Uniform Monolayer WS_2 on Reusable Au Foils. *Nat. Commun.* **2015**, *6*, 8569.

(9) Li, T.; Guo, W.; Ma, L.; Li, W.; Yu, Z.; Han, Z.; Gao, S.; Liu, L.; Fan, D.; Wang, Z.; Yang, Y.; Lin, W.; Luo, Z.; Chen, X.; Dai, N.; Tu, X.; Pan, D.; Yao, Y.; Wang, P.; Nie, Y.; et al. Epitaxial Growth of Wafer-Scale Molybdenum Disulfide

Semiconductor Single Crystals on Sapphire. *Nat. Nanotechnol.* **2021**, *16*, 1201–1207.

(10)Fu, J.-H.; Min, J.; Chang, C.-K.; Tseng, C.-C.; Wang, Q.; Sugisaki, H.; Li, C.; Chang, Y.-M.; Alnami, I.; Syong, W.-R.; Lin, C.; Fang, F.; Zhao, L.; Lo, T.-H.; Lai, C.-S.; Chiu, W.-S.; Jian, Z.-S.; Chang, W.-H.; Lu, Y.-J.; Shih, K.; et al. Oriented Lateral Growth of Two-Dimensional Materials on c-Plane Sapphire. *Nat. Nanotechnol.* **2023**, *18*, 1289–1294.

(11)Wang, J.; Xu, X.; Cheng, T.; Gu, L.; Qiao, R.; Liang, Z.; Ding, D.; Hong, H.; Zheng, P.; Zhang, Z.; Zhang, Z.; Zhang, S.; Cui, G.; Chang, C.; Huang, C.; Qi, J.; Liang, J.; Liu, C.; Zuo, Y.; Xue, G.; et al. Dual-Coupling-Guided Epitaxial Growth of Wafer-Scale Single-Crystal WS_2 Monolayer on Vicinal *a*-Plane Sapphire. *Nat. Nanotechnol.* **2022**, *17*, 33–38.

(12)Hoang, A. T.; Hu, L.; Kim, B. J.; Van, T. T. N.; Park, K. D.; Jeong, Y.; Lee, K.; Ji, S.; Hong, J.; Katiyar, A. K.; Shong, B.; Kim, K.; Im, S.; Chung, W. J.; Ahn, J.-H. Low-Temperature Growth of MoS_2 on Polymer and Thin Glass Substrates for Flexible Electronics. *Nat. Nanotechnol.* **2023**, *18*, 1439–1447.

(13)Chubarov, M.; Choudhury, T. H.; Hickey, D. R.; Bachu, S.; Zhang, T.; Sebastian, A.; Bansal, A.; Zhu, H.; Trainor, N.; Das, S.; Terrones, M.; Alem, N.; Redwing, J. M. Wafer-Scale Epitaxial Growth of Unidirectional WS_2 Monolayers on Sapphire. *ACS Nano* **2021**, *15*, 2532–2541.

(14)Liu, M.; Liao, J.; Liu, Y.; Li, L.; Wen, R.; Hou, T.; Ji, R.; Wang, K.; Xing, Z.; Zheng, D.; Yuan, J.; Hu, F.; Tian, Y.; Wang, X.; Zhang, Y.; Bachmatiuk, A.; Rümmeli, M. H.; Zuo, R.; Hao, Y. Periodical Ripening for MOCVD Growth of Large 2D Transition Metal Dichalcogenide Domains. *Adv. Funct. Mater.* **2023**, *33*, 2212773.

(15)Kang, K.; Xie, S.; Huang, L.; Han, Y.; Huang, P. Y.; Mak, K. F.; Kim, C.-J.; Muller, D.; Park, J. High-Mobility Three-Atom-Thick Semiconducting Films with Wafer-Scale Homogeneity. *Nature* **2015**, *520*, 656–660.

(16)Mun, J.; Park, H.; Park, J.; Joung, D.; Lee, S.-K.; Leem, J.; Myoung, J.-M.; Park, J.; Jeong, S.-H.; Chegal, W.; Nam, S.; Kang, S.-W. High-Mobility MoS_2 Directly Grown on Polymer Substrate with Kinetics-Controlled Metal–Organic Chemical Vapor Deposition. *ACS Appl. Electron. Mater.* **2019**, *1*, 608–616.

(17)Schaefer, C. M.; Caicedo Roque, J. M.; Sauthier, G.; Bousquet, J.; Hébert, C.; Sperling, J. R.; Pérez-Tomás, A.; Santiso, J.; del Corro, E.; Garrido, J. A. Carbon Incorporation in MOCVD of MoS_2 Thin Films Grown from an Organosulfide Precursor. *Chem. Mater.* **2021**, *33*, 4474–4487.

(18)Kim, T. S.; Dhakal, K. P.; Park, E.; Noh, G.; Chai, H.-J.; Kim, Y.; Oh, S.; Kang, M.; Park, J.; Kim, J.; Kim, S.; Jeong, H. Y.; Bang, S.; Kwak, J. Y.; Kim, J.; Kang, K. Gas-Phase Alkali Metal-Assisted MOCVD Growth of 2D Transition Metal Dichalcogenides for Large-Scale Precise Nucleation Control. *Small* **2022**, *18*, e2106368.

(19)Zhang, K.; Bersch, B. M.; Zhang, F.; Briggs, N. C.; Subramanian, S.; Xu, K.; Chubarov, M.; Wang, K.; Lerach, J. O.; Redwing, J. M.; Fullerton-Shirey, S. K.; Terrones, M.; Robinson, J. A. Considerations for Utilizing Sodium Chloride in Epitaxial Molybdenum Disulfide. *ACS Appl. Mater. Interfaces* **2018**, *10*, 40831–40837.

(20)Kim, H.; Ovchinnikov, D.; Deiana, D.; Unuchek, D.; Kis, A. Suppressing Nucleation in Metal-Organic Chemical Vapor Deposition of MoS_2 Monolayers by Alkali Metal Halides. *Nano Lett.* **2017**, *17*, 5056–5063.

(21)Cohen, A.; Patsha, A.; Mohapatra, P. K.; Kazes, M.; Ranganathan, K.; Houben, L.; Oron, D.; Ismach, A. Growth-Etch Metal-Organic Chemical Vapor Deposition Approach of WS_2 Atomic Layers. *ACS Nano* **2021**, *15*, 526–538.

(22)Park, J.-H.; Lu, A.-Y.; Shen, P.-C.; Shin, B. G.; Wang, H.; Mao, N.; Xu, R.; Jung, S. J.; Ham, D.; Kern, K.; Han, Y.; Kong, J. Synthesis of High-Performance Monolayer Molybdenum Disulfide at Low Temperature. *Small Methods* **2021**, *5*, e2000720.

(23)Zhu, J.; Park, J.-H.; Vitale, S. A.; Ge, W.; Jung, G. S.; Wang, J.; Mohamed, M.; Zhang, T.; Ashok, M.; Xue, M.; Zheng, X.; Wang, Z.; Hansryd, J.; Chandrakasan, A. P.; Kong, J.; Palacios, T. Low-Thermal-Budget Synthesis of Monolayer Molybdenum Disulfide for Silicon Back-End-of-Line Integration on a 200 Mm Platform. *Nat. Nanotechnol.* **2023**, *18*, 456–463.

(24)Wang, Q.; Li, N.; Tang, J.; Zhu, J.; Zhang, Q.; Jia, Q.; Lu, Y.; Wei, Z.; Yu, H.; Zhao, Y.; Guo, Y.; Gu, L.; Sun, G.; Yang, W.; Yang, R.; Shi, D.; Zhang, G. Wafer-Scale Highly Oriented Monolayer MoS_2 with Large Domain Sizes. *Nano Lett.* **2020**, *20*, 7193–7199.

(25)Wang, S.; Rong, Y.; Fan, Y.; Pacios, M.; Bhaskaran, H.; He, K.; Warner, J. H. Shape Evolution of Monolayer MoS_2 Crystals Grown by Chemical Vapor Deposition. *Chem. Mater.* **2014**, *26*, 6371–6379.

(26)Sebastian, A.; Pendurthi, R.; Choudhury, T. H.; Redwing, J. M.; Das, S. Benchmarking Monolayer MoS_2 and WS_2 Field-Effect Transistors. *Nat. Commun.* **2021**, *12*, 693.

(27)Dorow, C. J.; O'Brien, K. P.; Naylor, C. H.; Lee, S.; Penumatcha, A.; Hsiao, A.; Tronic, T.; Christenson, M.; Maxey, K.; Zhu, H.; Oni, A.; Alaan, U. S.; Gosavi, T. A.; Sen Gupta, A.; Bristol, R.; Clendenning, S.; Metz, M.; Avci, U. E. Advancing

Monolayer 2D NMOS and PMOS Transistor Integration From Growth to van Der Waals Interface Engineering for Ultimate CMOS Scaling. In *2021 Symposium on VLSI Technology*; IEEE, 2021; pp 1–2.

(28)Lin, Y.; Shen, P.-C.; Su, C.; Chou, A.-S.; Wu, T.; Cheng, C.-C.; Park, J.-H.; Chiu, M.-H.; Lu, A.-Y.; Tang, H.-L.; Tavakoli, M. M.; Pitner, G.; Ji, X.; McGahan, C.; Wang, X.; Cai, Z.; Mao, N.; Wang, J.; Wang, Y.; Tisdale, W.; et al. Contact Engineering for High-Performance N-Type 2D Semiconductor Transistors. In *2021 IEEE International Electron Devices Meeting (IEDM)*; IEEE, 2021; p 37.2.1-37.2.4.

(29)Shen, P.-C.; Su, C.; Lin, Y.; Chou, A.-S.; Cheng, C.-C.; Park, J.-H.; Chiu, M.-H.; Lu, A.-Y.; Tang, H.-L.; Tavakoli, M. M.; Pitner, G.; Ji, X.; Cai, Z.; Mao, N.; Wang, J.; Tung, V.; Li, J.; Bokor, J.; Zettl, A.; Wu, C.-I.; et al. Ultralow Contact Resistance between Semimetal and Monolayer Semiconductors. *Nature* **2021**, *593*, 211–217.

(30)Wan, Y.; Li, E.; Yu, Z.; Huang, J.-K.; Li, M.-Y.; Chou, A.-S.; Lee, Y.-T.; Lee, C.-J.; Hsu, H.-C.; Zhan, Q.; Aljarb, A.; Fu, J.-H.; Chiu, S.-P.; Wang, X.; Lin, J.-J.; Chiu, Y.-P.; Chang, W.-H.; Wang, H.; Shi, Y.; Lin, N.; et al. Low-Defect-Density WS_2 by Hydroxide Vapor Phase Deposition. *Nat. Commun.* **2022**, *13*, 4149.

(31)Jin, L.; Koester, S. J. High-Performance Dual-Gated Single-Layer WS_2 MOSFETs With Bi Contacts. *IEEE Electron Device Lett.* **2022**, *43*, 639–642.

(32)Li, M.-Y.; Hsu, C.-H.; Shen, S.-W.; Chou, A.-S.; Lin, Y. C.; Chuu, C.-P.; Yang, N.; Chou, S.-A.; Huang, L.-Y.; Cheng, C.-C.; Woon, W.-Y.; Liao, S.; Wu, C.-I.; Li, L.-J.; Radu, I.; Wong, H.-S. P.; Wang, H. Wafer-Scale Bi-Assisted Semi-Auto Dry Transfer and Fabrication of High-Performance Monolayer CVD WS_2 Transistor. In *2022 IEEE Symposium on VLSI Technology and Circuits (VLSI Technology and Circuits)*; IEEE, 2022; pp 290–291.

(33)Shi, X.; Li, X.; Guo, Q.; Gao, H.; Zeng, M.; Han, Y.; Yan, S.; Wu, Y. Improved Self-Heating in Short-Channel Monolayer WS_2 Transistors with High-Thermal Conductivity BeO Dielectrics. *Nano Lett.* **2022**, *22*, 7667–7673.

(34)Sun, Z.; Pang, C.-S.; Wu, P.; Hung, T. Y. T.; Li, M.-Y.; Liew, S. L.; Cheng, C.-C.; Wang, H.; Wong, H.-S. P.; Li, L.-J.; Radu, I.; Chen, Z.; Appenzeller, J. Statistical Assessment of High-Performance Scaled Double-Gate Transistors from Monolayer WS_2. *ACS Nano* **2022**, *16*, 14942–14950.

(35)Shi, X.; Li, X.; Guo, Q.; Zeng, M.; Wang, X.; Wu, Y. Ultrashort Channel Chemical Vapor Deposited Bilayer WS_2 Field-Effect Transistors. *Appl. Phys. Rev.* **2023**, *10*, 011405.

(36)Choi, S. H.; Kim, H.-J.; Song, B.; Kim, Y. I.; Han, G.; Nguyen, H. T. T.; Ko, H.; Boandoh, S.; Choi, J. H.; Oh, C. S.; Cho, H. J.; Jin, J. W.; Won, Y. S.; Lee, B. H.; Yun, S. J.; Shin, B. G.; Jeong, H. Y.; Kim, Y.-M.; Han, Y.-K.; Lee, Y. H.; et al. Epitaxial Single-Crystal Growth of Transition Metal Dichalcogenide Monolayers via the Atomic Sawtooth Au Surface. *Adv. Mater.* **2021**, *33*, e2006601.

(37)Zhou, S.; Liu, L.; Cui, S.; Ping, X.; Hu, D.; Jiao, L. Fast Growth of Large Single-Crystalline WS_2 Monolayers via Chemical Vapor Deposition. *Nano Res.* **2021**, *14*, 1659–1662.

(38)Yang, H.; Wang, Y.; Zou, X.; Bai, R.; Wu, Z.; Han, S.; Chen, T.; Hu, S.; Zhu, H.; Chen, L.; Zhang, D. W.; Lee, J. C.; Lu, X.; Zhou, P.; Sun, Q.; Yu, E. T.; Akinwande, D.; Ji, L. Wafer-Scale Synthesis of WS_2 Films with In Situ Controllable p-Type Doping by Atomic Layer Deposition. *Research* **2021**, *2021*, 9862483.
